# Analysis of the Genetic Stability of Insect and Herbicide Resistance Genes in Transgenic Rice Lines: A Laboratory and Field Experiment

**DOI:** 10.1186/s12284-023-00624-5

**Published:** 2023-02-13

**Authors:** Yue Sun, Zhongkai Chen, Huizhen Chen, Chunlei Wang, Bai Li, Lu Qin, Xiaoli Lin, Yicong Cai, Dahu Zhou, Linjuan Ouyang, Changlan Zhu, Haohua He, Xiaosong Peng

**Affiliations:** 1 Key Laboratory of Crop Physiology, Ecology, and Genetic Breeding, Ministry of Education /College of Agronomy, Jiangxi Agricultural University, Nanchang, Jiangxi China; 2 Hainan Yazhou Bay Seed Laboratory, Sanya, Hainan China; 3 College of Agronomy, Hunan Agricultural University, Changsha, Hunan China; 4 Pingxiang Center for Agricultural Sciences and Technology Research, Pingxiang, Jiangxi China

**Keywords:** *Bacillus thuringiensis*, Genetic stability, Transgenic rice, Insect resistance, Herbicide resistance

## Abstract

A lack of stability in the expression of *Bacillus thuringiensis* genes (*CRY*) and the dialaninophosphate resistance gene (*BAR*) in transgenic rice plants can lead to the loss of important characters. The genetic stability of transgenic expression in high-generation lines is thus critically important for ensuring the success of molecular breeding efforts. Here, we studied the genetic stability of resistance to insect pests and herbicides in transgenic rice lines at the molecular and phenotypic levels in a pesticide-free environment. Southern blot analysis, real-time polymerase chain reaction, and enzyme-linked immunosorbent assays revealed high stability in the copy numbers and expression levels of *CRY1C*, *CRY2A*, and *BAR* in transgenic lines across different generations, and gene expression levels were highly correlated with protein expression levels. The insecticide resistance of the transgenic rice lines was high. The larval mortality of *Chilo suppressalis* was 50.25% to 68.36% higher in transgenic lines than in non-transgenic control lines. Percent dead hearts and percent white spikelets were 16.66% to 22.15% and 27.07% to 33.47% lower in transgenic lines than in non-transgenic control lines, respectively. The herbicide resistance of the transgenic rice lines was also high. The bud length and root length ranged were 2.53 cm to 4.20 cm and 0.28 cm to 0.73 cm higher in transgenic lines than in non-transgenic control lines in the budding stage, respectively. Following application of the herbicide Basta, the chlorophyll content of the transgenic lines began to recover 2 d later in the seedling and tillering stages and 3 d later in the booting and heading stages, by contrast, the chlorophyll content of the non-transgenic lines did not recover and continued to decrease. These findings revealed high genetic stability of the resistance to insect pests and herbicides across several generations of transgenic rice regardless of the genetic background.

**Supplementary Information:**

The online version contains supplementary material available at 10.1186/s12284-023-00624-5.

## Introduction

Rice is the world’s most important staple food crop. Rapid and continuous changes in the environment driven by anthropogenic activities have increased the intensity and frequency at which rice plants are exposed to abiotic and biotic stresses, such as heat and cold stress, drought, heavy metals, herbicides, pests, and diseases, and this has accelerated rice yield losses (Li et al. 2016). *Chilo suppressalis* is an economically significant pest that can induce substantial damage to rice plants, and it has been estimated to be responsible for losses that amount to 10–30% of total rice production (Jian et al. 2014). *Bacillus thuringiensis* (Bt) proteins are highly effective biocides against insect pests, and they function by destroying the midgut cells (Vaeck et al. 1987). The Bt toxin is highly specific and thus non-toxic to various animals, such as reptiles, birds, and mammals (including humans) (Stewart et al. 1996). The most commonly used *Bt* genes in transgenic crops include *CRY1Ab/Ac*, *CRY1C*, and *CRY2A* (Wünn et al. 1996; Chen et al. 2005; Tang et al. 2006). A biosafety certificate was issued for MH63 (*CRY1Ab/Ac*) in 2009, and the commercial production of MH63 (*CRY1Ab/Ac*) began in China that same year; several studies have examined transgenic rice with the *CRY1Ab/Ac* gene and its derivatives (Chen et al. 2011). Efforts to test the potential utility of the transgenic rice lines MH63 (*CRY1C*) and MH63 (*CRY2A*) in China were accelerated in 2021, and the genetic stability of key traits in transgenic rice is critically important for evaluating the ability of transgenic traits to make significant contributions to ongoing breeding programs (Gahakwa et al. 2000). The genetic stability of transgenic traits is typically evaluated over at least two generations to ensure the success of the breeding of commercially important varieties using genetic engineering approaches.

Following the introduction of foreign genes into recipient cells, callus induction, adventitious bud differentiation, adventitious root formation, and other culture processes are required for asexual propagation. In the sexual stage, plants germinate, grow, and flower, and this is followed by meiosis, pollination, fertilization, zygote formation, and embryo development; various vegetative growth and reproductive growth processes also take place in this stage (Azhakanandam et al. 2000). Next, foreign genes are transferred to cultivars with desirable traits through sexual hybridization (i.e., transfer process), and these cultivars are then used to cultivate new varieties (Bavage et al. 2002). These processes, especially the transfer process, challenge the genetic stability of foreign genes. At the molecular level, the integration site, gene fragment size, copy number, methylation, repeat sequences, trans-inactivation, and co-inhibition of foreign genes can all have an effect on their stable inheritance during asexual and sexual reproduction (Fernandez et al. 2009; Marjanac et al. 2009; Mette et al. 2000; Que et al. 1997). Polymerase chain reaction (PCR) screening of marker genes or reporter genes is one of the main methods used to detect foreign genes. Although rapid and convenient, this method is prone to producing false positives. PCR can be used to determine whether the target gene has been integrated into the chromosomes of recipient cells; however, this method is also prone to the effects of DNA contamination, and this increases the difficulty of detecting multilocus insertions. Molecular hybridization is currently the most effective method for determining whether foreign genes have been integrated into plant chromosomes. The copy number and insertion mode of exogenous genes can be determined using southern hybridization analysis (Maghuly et al. 2007), and the expression level of foreign genes can be determined using real-time PCR (Zhang et al. 2017). The proteins expressed in transgenic plants can be identified using enzyme-linked immunosorbent assays (ELISAs) in the event that the exogenous genes are protein-coding genes. Robust assessments of genetic stability require the accurate identification of foreign genes (Xu et al. 2018).

The development of genetically stable high-generation lines is essential for generating transgenic rice hybrids with high yield and quality and resistance to insects, diseases, and herbicides. Here, we characterized the insect and herbicide resistance of newly bred *Bt*-transgenic rice parental lines with the *CRY1C*, *CRY2A*, and *BAR* genes in different genetic backgrounds. Specifically, our aim was to assess the genetic stability of *Bt*-transgenic rice lines from different generations at the DNA, RNA, and protein levels as well as their insect resistance and herbicide resistance phenotypes.

## Results

### Genetic Background Analysis

**Fig. 1:**
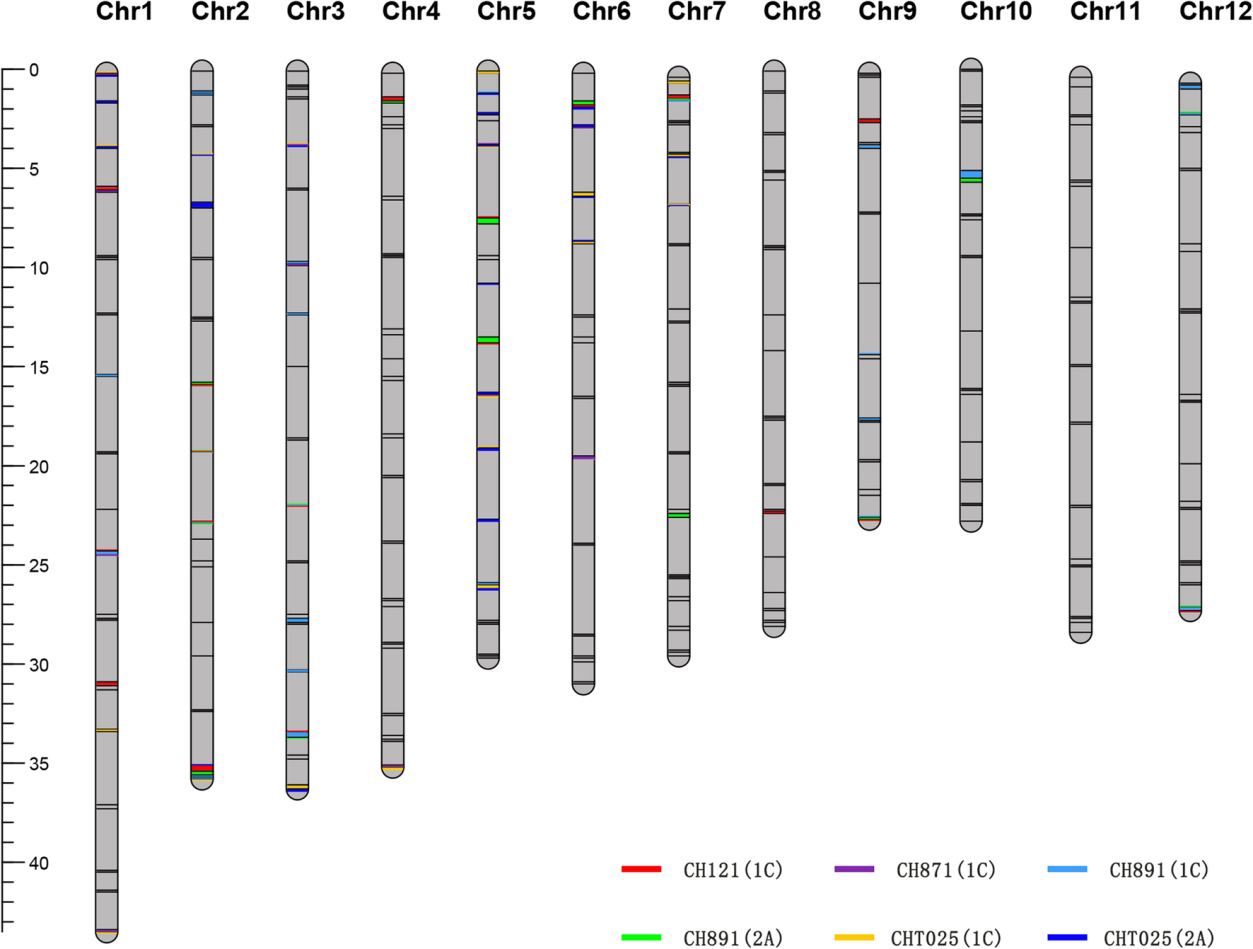
The linkage map of CH121(1C), CH871(1C), CH891(1C), CH891(2A), CHT025(1C), and CHT025(2A). The red, purple, sky blue, green, yellow, and blue bars indicate the Minghui 63 chromosomal segments in CH121(1C), CH871(1C), CH891(1C), CH891(2A), CHT025(1C), and CHT025(2A), respectively. The scale of the ruler indicates the genetic distance in “cM.”

We analyzed the response rate of the genetic background using six BC_4_F_8_ and BC_4_F_9_ populations. A total of 512 molecular markers were used for genotyping, and the linkage map of SSR fingerprint markers was generated (Fig. 1). A total of 31, 10, 23, 11, 21, and 25 polymorphic markers between CH121(1C), CH871(1C), CH891(1C), CH891(2A), CHT025(1C) and CHT025(2A) and their parents were identified, and the percentage of polymorphic markers was 6.05%, 1.95%, 4.49%, 2.15%, 4.10%, and 4.88%, respectively. The low number of polymorphic markers identified suggests that the genetic background of the six transgenic recovered lines was similar to that of their recurrent parents. The response rate of the actual genetic background of CH121(1C), CH871(1C), CH891(1C), CH891(2A), CHT025(1C), and CHT025(2A) was 96.97%, 99.02%, 97.75%, 98.93%, 97.95%, and 97.56%, respectively (Additional file 1: Table S1). The actual response rate of the genetic background was higher than the theoretical response rate of the genetic background.

### PCR and Southern Blot Analysis

**Fig. 2:**
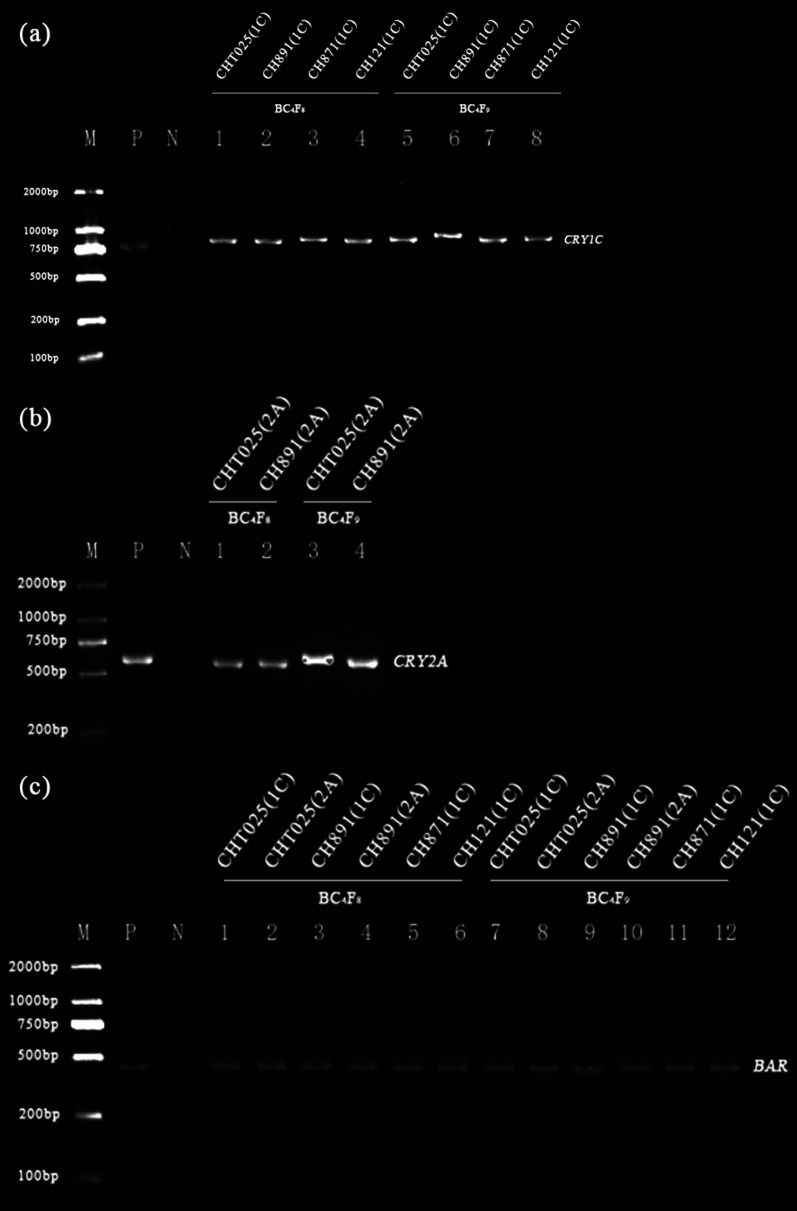
Marker-assisted selection of target genes in the transgenic restorer lines. M indicates marker; P indicates the donor parent Minghui 63; N indicates the receptor parent (recurrent parent); **a** 1–8 correspond to the transgenic *CRY1C* gene-resistant restorer lines. **b** 1–4 correspond to the transgenic *CRY2A* gene-resistant restorer lines. **c** 1–12 correspond to the transgenic *BAR* gene-resistant restorer lines

**Fig. 3:**
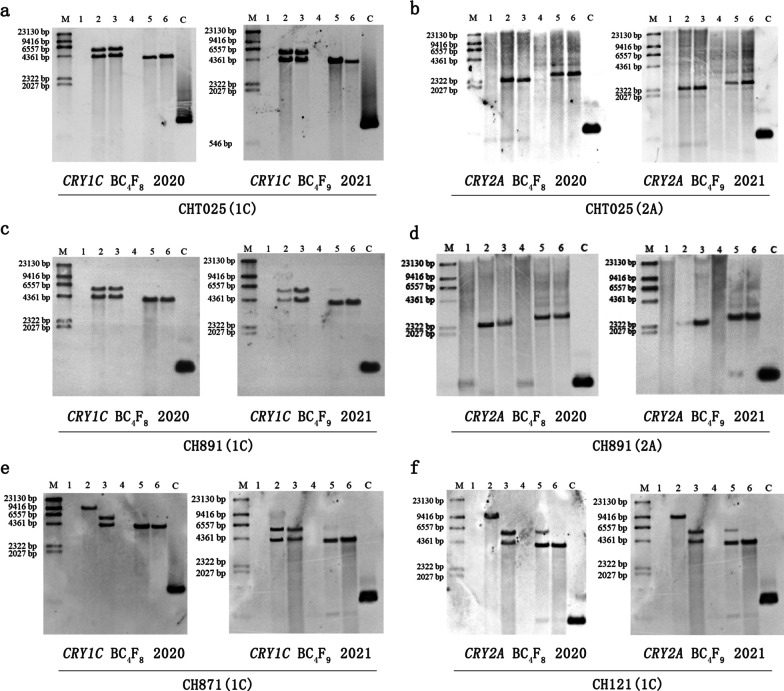
Southern blot analysis of the total genomic DNA of BC_4_F_8_ and BC_4_F_9_ transgenic plants. Southern blot of **a** CHT025(1C), **b** CHT025(2A), **c** CH891(1C), **d** CH891(2A), **e** CH871(1C), and **f** CH121(1C) in BC_4_F_8_ and BC_4_F_9_ transgenic plants. The DNA samples were digested with *Nco*I and *Hind*III and hybridized with the prepared radioactive probe (*Nco*I and *Hind*III restriction sites are absent within the T-DNA region). The size of the probe was 4.3 and 5.5 kb for *Nco*I and 4.1 kb for *Hind*III and was a fragment of the *CRY1C* gene. The DNA samples were digested with *Bam*HI and *Eco*RI and hybridized with the prepared radioactive probe (*Bam*HI and *Eco*RI restriction sites are absent within the T-DNA region). The size of the probe was 2.5 kb for *Bam*HI and 3.5 kb for *Eco*RI and was a fragment of the *CRY2A* gene. M: DNA marker; 1 and 4: negative control; 2 and 5: positive control; 3 and 6: BC_4_F_8_ and BC_4_F_9_ transgenic plants, respectively; C: PCR product

**Fig.4:**
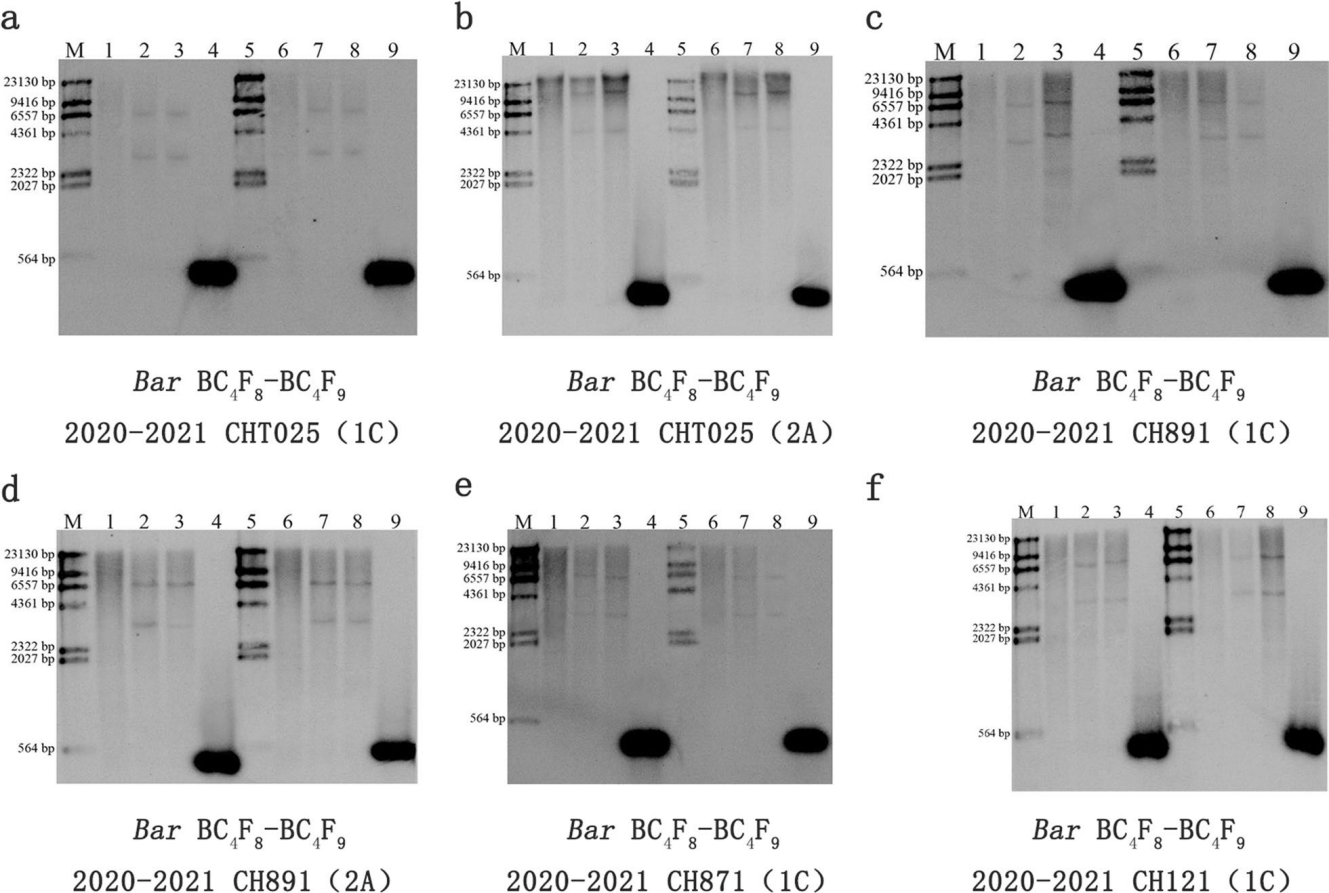
Southern blot analysis of the total genomic DNA of BC_4_F_8_ and BC_4_F_9_ transgenic plants. Southern blot of **a** CHT025(1C), **b** CHT025(2A), **c** CH891(1C), **d** CH891(2A), **e** CH871(1C), and **f** CH121(1C) in BC_4_F_8_ and BC_4_F_9_ transgenic plants. The DNA samples were digested with *SmaI* and hybridized with the prepared radioactive probe (*SmaI* restriction sites are absent within the T-DNA region). The size of the probe was 6.8 and 3.0 kb for *SmaI* and was a fragment of the *BAR* gene. M: DNA marker; 1 and 6: negative control; 2 and 7: positive control; 3 and 8: BC_4_F_8_ and BC_4_F_9_ transgenic plants, respectively; 4 and 9: PCR product. 1–4: southern blot analysis BC_4_F_8_ in 2020; 1–4: southern blot analysis BC_4_F_9_ in 2021

The six high-generation transgenic rice lines from the BC_4_F_8_ and BC_4_F_9_ populations were shown to be transgene-positive according to PCR analysis. The 799-bp, 600-bp, and 479-bp molecular weight bands were amplified from the *CRY1C*, *CRY2A*, and *BAR* genes in the six transgenic lines, respectively, via PCR (Fig. 2). We characterized the integration sites of the *CRY1C* and *CRY2A* genes with variable copy numbers (Fig. 3). Southern blot analysis with the *CRY1C* gene revealed transgenes with two copies of the transforming DNA, and these transgenes were also consistently detected in the progeny analysis. *Nco*I and *Hind*III enzymes and uncut genomic DNA from transgenic plants showed different banding patterns at high molecular weights. Southern blot analysis of the *CRY2A* gene revealed a single copy of the transforming DNA in different generations and lines. *Bam*HI and *Eco*RI enzymes and uncut genomic DNA from transgenic plants showed different banding patterns at high molecular weights. The copy numbers of target genes were analyzed using Southern blot (Fig. 4). The Southern blot analysis with the *BAR* gene revealed two bands for *Sma*I enzyme-digested samples, indicating the presence of two copies of target genes in the rice genome. Southern blot-positive plants containing fragments consistent with the expected size of the *CRY1C*, *CRY2A*, and *BAR* genes were used in analyses of gene and protein expression and bioassay activity.

### Analysis of Gene and Protein Expression Patterns

**Fig. 5:**
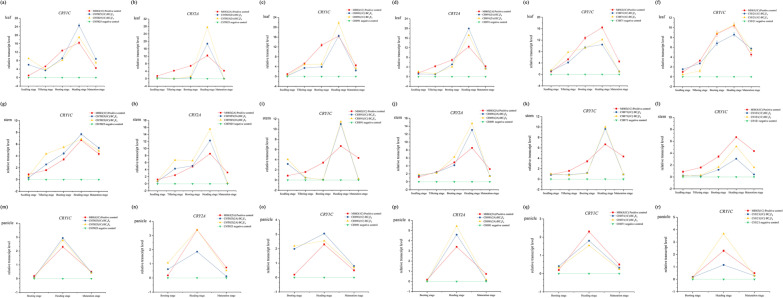
The relative expression levels of *CRY1C* and *CRY2A* were determined using real-time PCR. All tests were conducted in three replicates. The relative expression of *CRY1C* and *CRY2A* in the leaves (**a**–**f**), stem (**g**–**l**), and panicles (**m**–**r**) of six transgenic rice lines was measured at different developmental stages. *Osactin1* was used as an internal control. Values are mean ± standard error. The red line indicates the donor parent (positive control), the blue line indicates the high-generation transgenic rice line (BC_4_F_8_), the yellow line indicates the high-generation transgenic rice line (BC_4_F_9_), and the green line indicates the receptor parent (negative control)

**Fig.6:**
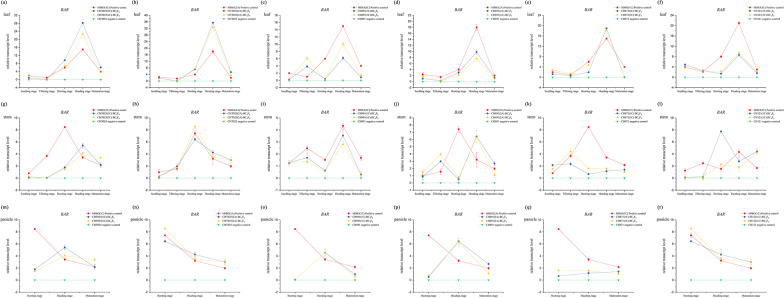
The relative expression levels of *BAR* were determined using real-time PCR. All tests were conducted in three replicates. The relative expression of *BAR* in the leaves (**a**–**f**), stem (**g**–**l**), and panicles (**m**–**r**) of six transgenic rice lines was measured at different developmental stages. *Osactin1* was used as an internal control. Values are mean ± standard error. The red line indicates the donor parent (positive control), the blue line indicates the high-generation transgenic rice line (BC_4_F_8_), the yellow line indicates the high-generation transgenic rice line (BC_4_F_9_), and the green line indicates the receptor parent (negative control)

The relative expression levels of *CRY1C*, *CRY2A*, and *BAR* in fresh leaves, stems (at the seedling, tillering, booting, heading, and filling stages), and endosperm (at the booting, heading, and filling stages) of the high-generation transgenic rice lines (BC_4_F_8_ and BC_4_F_9_) were measured. The expression of *CRY1C* and *CRY2A* was significantly higher in different tissues and at developmental stages in transgenic rice lines (Additional file 1: Table S2) compared with their non-transgenic parents (Additional file 1: Table S3). In various generations of all strains, the expression of *CRY1C* first increased and then decreased from the tillering stage to maturity, and its expression was highest at the heading stage. This gene was also expressed stably in the leaf, stem, and panicle (Fig. 5). The expression of *BAR* was significantly higher in different tissues and at developmental stages in transgenic rice lines (Additional file 1: Table S4) compared with their non-transgenic parents (Additional file 1: Table S5). In the leaves of all transgenic lines, the expression of *BAR* first increased and then decreased, and its expression was highest at the heading stage. However, the expression of *BAR* in CH871(1C) was highest in the stem and panicle at the tillering stage, and the expression of *BAR* was highest in the stem and panicle at the booting stage in CHT025(2A) and CH121(1C). The genetic background had an effect on the relative expression levels of *BAR*. The relative expression levels of *BAR* also varied among generations, indicating that the genetic environment has an effect on *BAR* expression (Fig. 6).

**Fig. 7:**
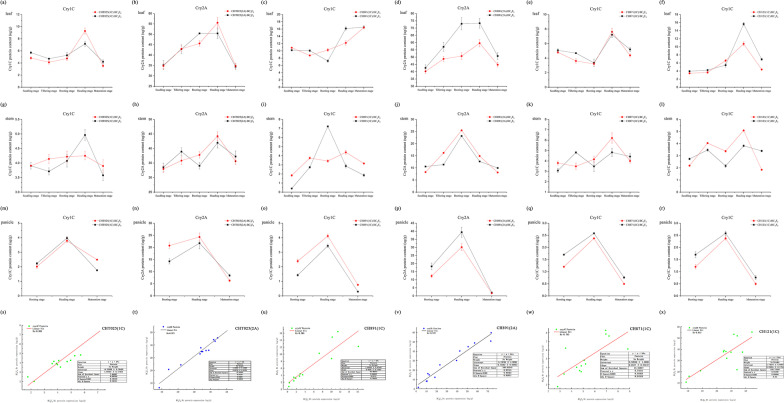
The Cry1C and Cry2A content was determined using ELISAs. The correlations between the content of Cry1C and Cry2A in transgenic rice for the two years of the field trials are shown. All tests were conducted with three replicates. The Cry1C and Cry2A content in (**a**–**f**) leaves, (**g**–**l**) stems, and (**m**–**r**) panicles of rice plants at different developmental stages. Values are mean ± standard error. Scatter plot of **s** CHT025(1C), **t** CHT025(2A), **u** CH891(1C), **v** CH891(2A), **w** CH871(1C), and **x** CH121(1C) in leaves, stems, and panicles at different developmental stages

**Fig.8:**
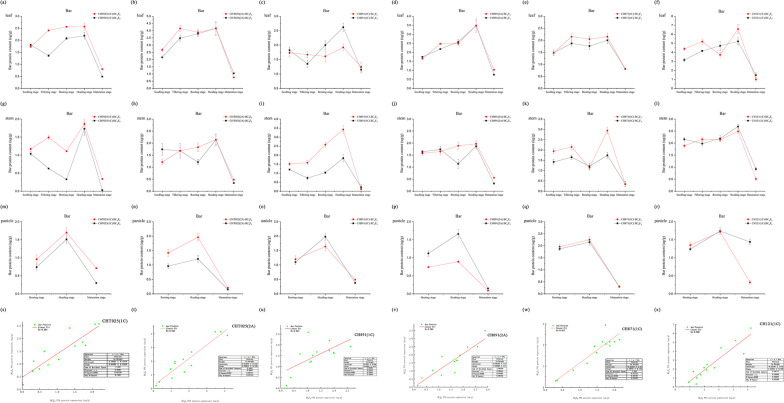
The Bar content was determined using ELISAs. The correlations between the content of Bar in transgenic rice for the two years of the field trials are shown. All tests were conducted with three replicates. The Bar content in (**a**–**f**) leaves, (**g**–**l**) stems, and (**m**–**r**) panicles of rice plants at different developmental stages. Values are mean ± standard error. Scatter plot of **s** CHT025(1C), **t** CHT025(2A), **u** CH891(1C), **v** CH891(2A), **w** CH871(1C), and **x** CH121(1C) in leaves, stems, and panicles at different developmental stages

The content of Cry1C and Cry2A in the six transgenic rice lines at various developmental stages and in several tissues was measured via ELISAs (Additional file 1: Table S6, Fig. 7). The concentration of Cry1C in the four transgenic lines ranged from 3.21 µg/g leaf fresh weight in the BC_4_F_9_ line of CH871(1C) at the booting stage to 16.16 µg/g leaf fresh weight in the BC_4_F_9_ line of CH871(1C) at the heading stage; the concentration of Cry2A ranged from 34.39 µg/g leaf fresh weight in the BC_4_F_9_ line of CHT025(2A) at the maturation stage to 73.33 µg/g leaf fresh weight in the BC_4_F_9_ line of CH891(2A) at the heading stage. The expression of Cry2A was higher than that of Cry1C in leaf tissue (Fig. 7a–f); the same pattern was also observed in the stem (Fig. 7g–l). The concentration of Cry1C decreased as plants transitioned from vegetative growth and reproductive growth. In the panicle, expression levels of Cry1C were low (Fig. 7m–r). Expression patterns of proteins and genes inferred from ELISAs and real-time PCR, respectively, were similar, and the expression of Cry1C and Cry2A was highest at the heading stage. Correlations between the expression levels of genes and proteins were high, with the exception of CH871(1C) (Rs = 0.719) (Fig. 7s–x). The content of the Bar protein in the six transgenic rice lines in several tissues and at various stages was determined using ELISA (Additional file 1: Table S7, Fig. 8). The expression of Bar was consistent across various generations (BC_4_F_8_ and BC_4_F_9_); the content of Bar first increased and then decreased, and its expression was highest at the heading stage. In the six transgenic lines, the concentration of Bar ranged from 0.49 µg/g to 6.58 µg/g leaf fresh weight and from 0.03 µg/g to 3.42 µg/g stem fresh weight (Fig. 8a–f). The expression pattern of the Bar protein first increased and then decreased as development progressed in both the leaves and stems; its expression was highest at the heading stage (Fig. 8g–l). The expression of the Bar protein was relatively stable during vegetative growth and reproductive growth (Fig. 8m–r). The expression patterns of the Bar protein and the *BAR* gene inferred from ELISA and real-time PCR, respectively, were basically consistent, and the expression of Bar was highest at the heading stage. Correlations between the expression of genes and proteins were high, with the exception of CH891(1C) (Fig. 8s–x).

### Resistance of Transgenic Rice Lines to *C. suppressalis* in the Laboratory and the Field

**Fig. 9:**
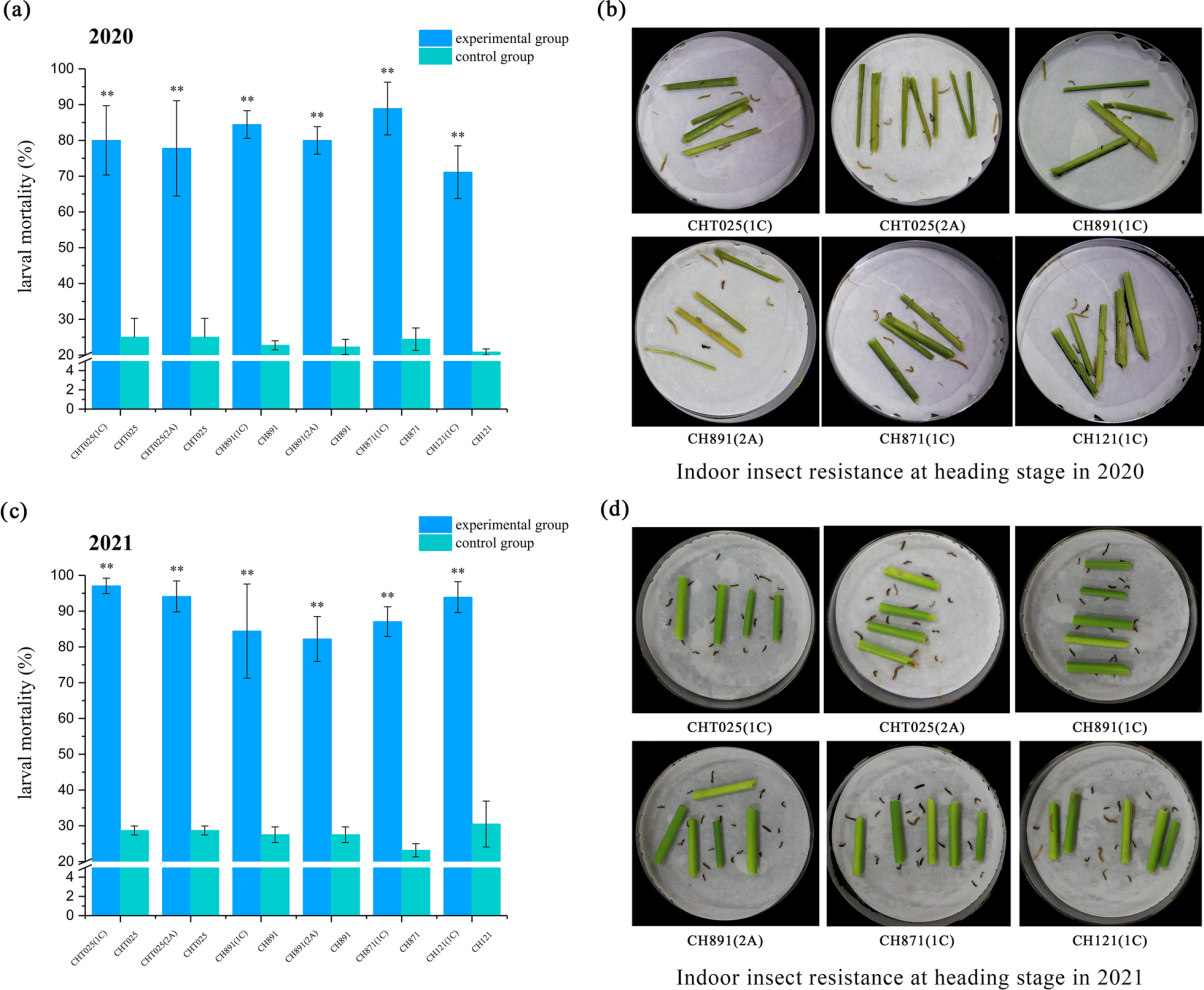
Insect resistance of BC_4_F_8_ and BC_4_F_9_ transgenic plants in the laboratory. Larval mortality of *C. suppressalis* in laboratory bioassays in 2020 (**a**) and 2021 (**c**). All tests were conducted with ten replicates, and one replicate comprised 20 s instar larvae. Values are mean ± standard error. Data followed by different lowercase letters denote significant differences between the mortality (%) rates of *C. suppressalis* at the 5% level according to least significant difference tests. Laboratory insect feeding tests of the stems of CHT025T(1C), CHT025T(2A), CH891T(1C), CH891T(1C), CH871T(1C), and CH121T(1C) at the heading stage in 2020 (**b**) and 2021 (**d**)

**Fig. 10:**
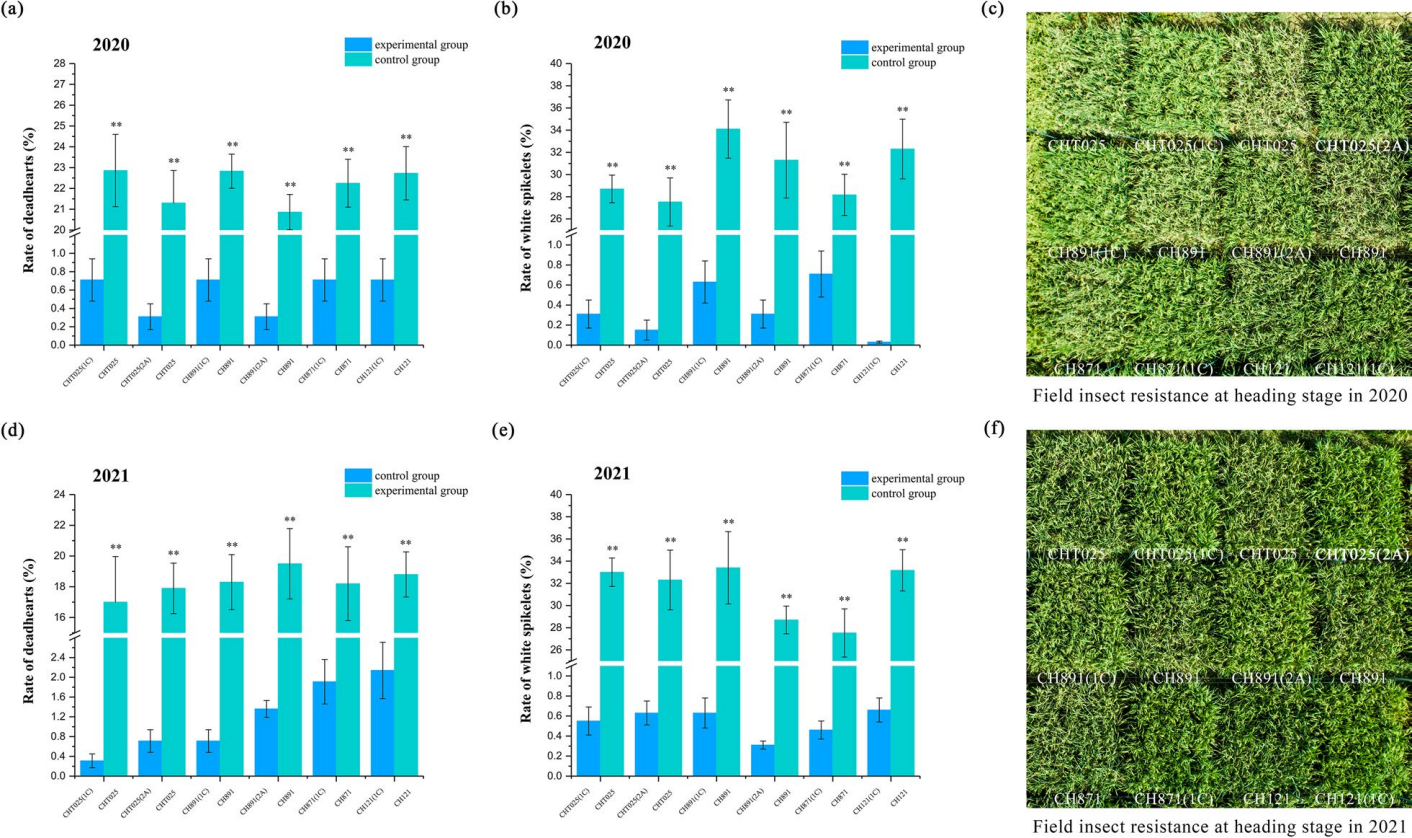
Insect resistance of BC_4_F_8_ and BC_4_F_9_ transgenic plants in the field. Percent dead hearts (%) in field trials in 2020 (**a**) and 2021 (**d**). Percent white spikelets (%) in field trials in 2020 (**b**) and 2021 (**e**). There were three replicates for all tests. Values are mean ± standard error. Data followed by different lowercase letters denote significant differences between mortality (%) rates of *C. suppressalis* at the 5% level according to least significant difference tests. Insecticide resistance at the heading stage of CHT025T(1C), CHT025T(2A), CH891T(1C), CH891T(1C), CH871T(1C), and CH121T(1C) in 2020 (**c**) and 2021 (**f**)

The resistance of the six transgenic plant lines and recurrent parents to *C. suppressalis* was tested in the laboratory (*P* < 0.05, Additional file 1: Table S8) and field (*P* < 0.05, Additional file 1: Table S9), and larval mortality, percent dead hearts, and percent white spikelets were determined. Stems at the heading stage (when borer damage is most common) were used for indoor insect-feeding tests. The mortality rate of *C. suppressalis* ranged from 71.11% to 88.89% and from 82.22% to 97.06% on the stems of the transgenic lines at the heading stage in 2020 and 2021; the transgenic plants thus showed high insect resistance (Fig. 9). In 2020, the percent dead hearts of the recurrent parents ranged from 20.86% to 22.86%, indicating that they were damaged to various degrees by *C. suppressalis*; however, the six transgenic lines only experienced slight damage (0.31–0.71%) from *C. suppressalis* (Fig. 10a). Recurrent parents were also extensively damaged by *C. suppressalis* at the heading stage, and the percent white spikelets ranged from 27.53% to 34.10%; however, the six transgenic lines only experienced slight damage (0.03–0.71%) from *C. suppressalis* (Fig. 10b). In 2020, the insect resistance of the six transgenic lines was similar and significantly (*P* < 0.01) higher than that of the recurrent parents (Fig. 10c). The percent dead hearts of the recurrent parents ranged from 17.00% to 19.50%, indicating that they were damaged to various degrees by *C. suppressalis*, and transgenic lines only experienced slight damage (0.31–2.14%) (Fig. 10d). Recurrent parents were also severely damaged by *C. suppressalis* at the heading stage, and the percent white spikelets ranged from 27.53% to 34.40%; the six transgenic lines only experienced slight damage (0.31–0.66%) by *C. suppressalis* (Fig. 10e). The damage induced to the recurrent parents by *C. suppressalis* might stem from differences in the abundances of insect pests across the two years of the experiments. Overall, resistance to insect pests was stable (Fig. 10f).

### Herbicide Resistance of Transgenic Rice Lines in the Laboratory and Field

**Fig. 11:**
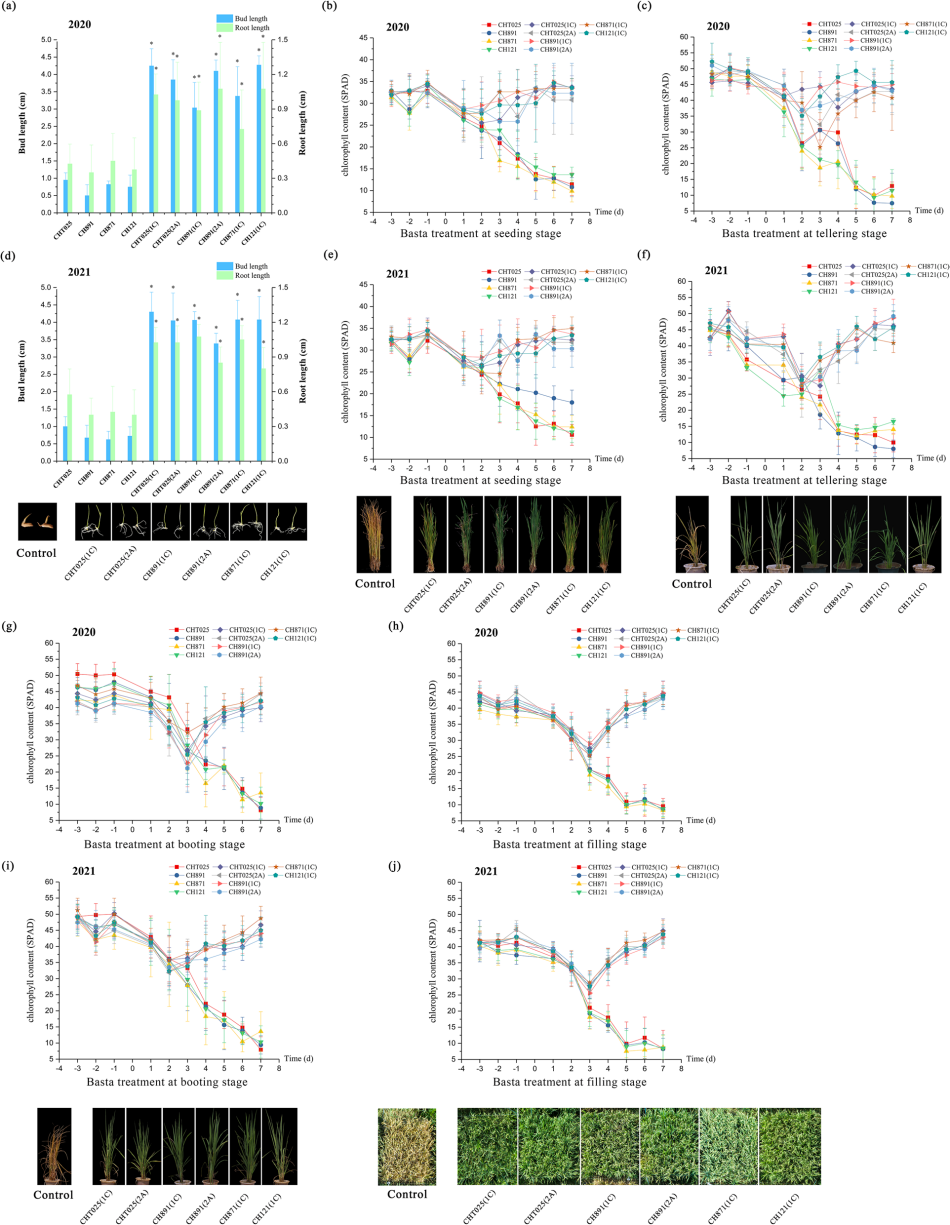
Herbicide resistance of BC_4_F_8_ and BC_4_F_9_ transgenic plants in the field. The bud length (cm) and root length (cm) were measured in a hydroponic system at the budding stage in 2020 (**a**) and 2021 (**d**). The chlorophyll content (SPAD) of rice plants at the seedling stage in field trials in 2020 (**b**) and 2021 (**e**). The chlorophyll content (SPAD) of rice plants at the tillering stage in field trials in 2020 (**c**) and 2021 (**f**). The chlorophyll content (SPAD) of rice plants at the booting stage in field trials in 2020 (**g**) and 2021 (**i**). The chlorophyll content (SPAD) of rice plants at the booting stage in field trials in 2020 (**h**) and 2021 (**j**). There were five replicates for all tests. Values are mean ± standard error. Data followed by different lowercase letters denote significant differences in bud length, root length, and chlorophyll content at the 5% level according to least significant difference tests

Weed management is essential for maximizing the productivity of rice cultivation. We tested the herbicide resistance of transgenic rice plants from five growth stages (*P* < 0.05, Additional file 1: Tables S10, S11). The herbicide Basta was used to evaluate the herbicide resistance of *BAR*-transgenic rice at the bud stage. Basta application strongly inhibited the growth of non-transgenic lines, and no increases in root length and shoot length were observed at a Basta concentration of 10 mg/L. By contrast, the resistance of transgenic rice with the *BAR* gene to Basta was high, and no inhibition of germination was evident under 10 mg/L Basta application at the budding stage in 2020 and 2021 (Fig. 11a, d). In field experiments in 2020 and 2021, we first examined herbicide resistance at the seedling and tiller stages. After Basta application for 7 d, non-transgenic lines had withered and yellow leaves, and their growth stagnated; they eventually died within 12 d of Basta application (Fig. 11b, c, e, f). However, the chlorophyll content in the leaves of the six transgenic rice lines began to return to their normal values 2 d after Basta application, and herbicide resistance at the booting stage and grain filling stage slightly differed. Thereafter, the chlorophyll content in the leaves began to return to normal (Fig. 11g–j). After 7 d of Basta application, *BAR*-transgenic rice could still grow normally in each growth period, and leaves showed no signs of dryness and yellowing, indicating that they had high herbicide resistance; no weeds were observed growing in the field (Fig. 11). The results suggested that the cultivation of *BAR*-transgenic rice along with Basta application might be an effective strategy for overcoming the deleterious effects of weeds on rice cultivation.

### Grain Yield Performance

**Fig. 12:**
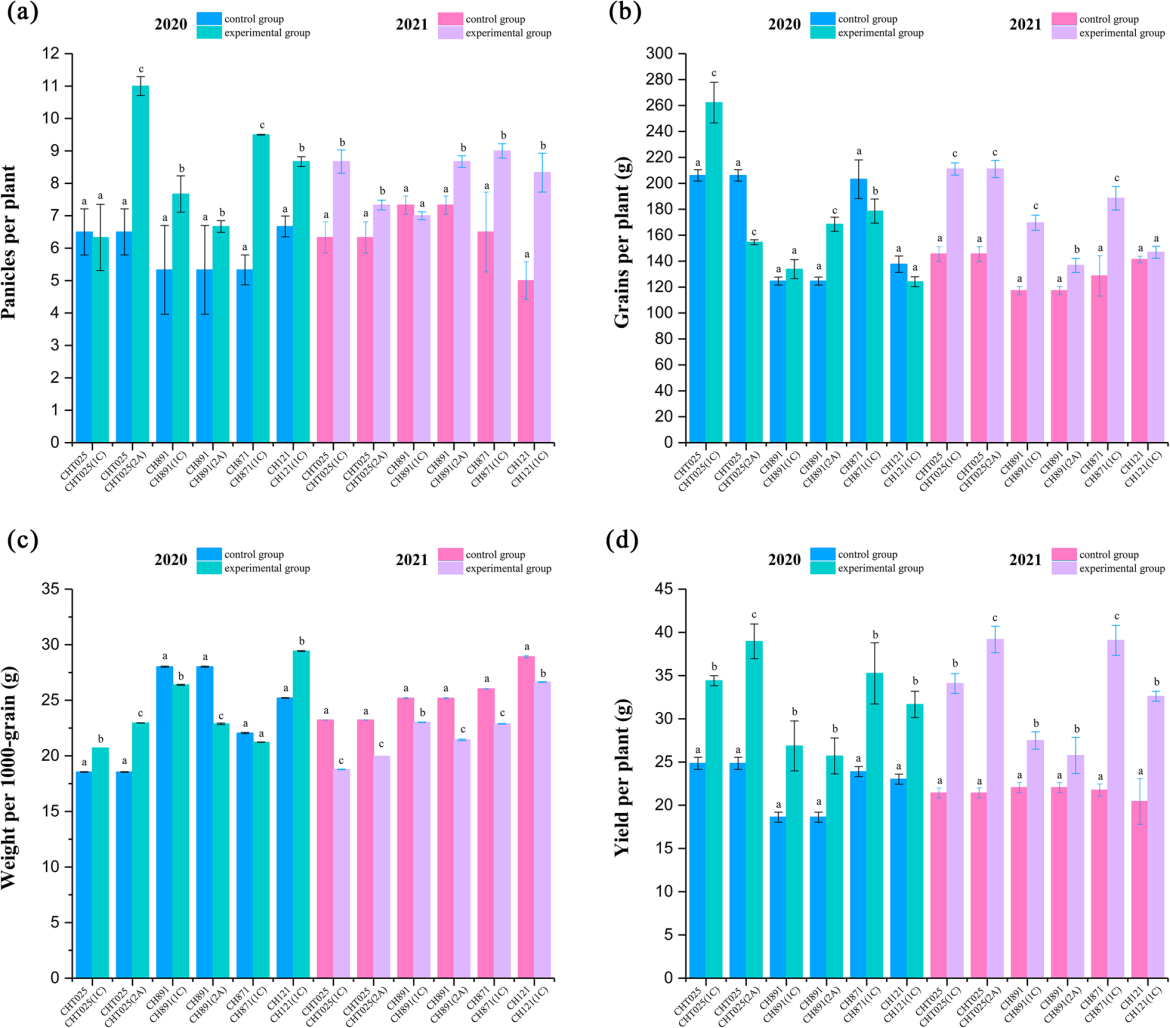
Yields and relative traits of BC_4_F_8_ and BC_4_F_9_ transgenic plants in the field. Panicles per plant, Grains per plant, Weight per 1000-grain and yield per plant in field trials in 2020 and 2021 (**a**-**d**). Percent white spikelets (%) in field trials in 2020 (**b**) and 2021 (**e**). There were three replicates for all tests. Values are mean ± standard error. Data followed by different lowercase letters denote significant differences between yields and relative traits at the 5% level according to least significant difference tests

Different years and different genotype of Bt-transgenic rice lines showed different yield performances (*P* < 0.05, Additional file 1: Table S12). In 2020, yields per plant of the six Bt-transgenic rice lines (CHT025(1C), CHT025(2A), CH891(1C), CH891(2A), CH871(1C), CH121(1C)) were around 34.41 g, 38.96 g, 26.87 g, 25.70 g, 35.26 g, and 31.66 g, respectively. In 2021, the corresponding lines were around 34.08 g, 39.18 g, 27.50 g, 25.76 g, 39.07 g, and 32.61 g, respectively. There was no significant difference in the yield traits of the same Bt-transgenic rice lines in different years. Therefore, the yield traits showed good genetic stability. On the other hand, the six Bt-transgenic rice lines had higher yields than their respective non-transgenic counterparts in 2020 and 2021, and the yield advantages were mainly due to the more grain per panicle and higher weight per 1000-grain of Bt-transgenic rice lines comparing with their respective non-transgenic counterparts. Genotype had significant effects on yield and relative traits of Bt-transgenic rice line. However, year had significant effects, in addition to some lines of grains per panicle (Fig. 12).

## Discussion

In our study, all transgenic rice lines showed higher resistance to insect pests and herbicides compared with non-transgenic control lines under pesticide-free conditions. Our findings provided new insights into the molecular basis of the stable inheritance of these traits. First, the copy number of the foreign genes in all transgenic rice lines remained unchanged during the two years of the field experiments compared with non-transgenic control lines. Previous studies have indicated that multiple heterologous transgenes are stably inherited in diverse genetic backgrounds (Beaujean et al. 1998; Scott et al. 1998). Second, the expression patterns of exogenous genes and proteins were similar among different transgenic lines. Previous studies have shown that the expression patterns of the *CRY* and *BAR* genes are similar under different genetic backgrounds (Cao et al. 1992; Chen et al. 1998; Yan et al. 2007; Zhou et al. 2016), but the correlations between gene and protein expression varied among genes (Peach and Velten 1991; Datta et al. 1998). Third, the higher expression of *Bt* genes and proteins in the leaves compared with the stem and panicle of the transgenic rice lines might stem from differences in the location of the tissues on the plant (Fearing et al. 1997; Kranthi et al. 2005). There was a strong correlation between the expression of exogenous genes and proteins, but the external environment had a greater effect on the expression of proteins, which explains why protein expression differed between the two years of the experiment (Jiang et al. 2014a, b). Ubiquitin promoter expression varied in different tissues and was generally higher in leaves (Tang et al. 2006). Meanwhile, in maize and rice, where starch is the main storage substance, the expression of exogenous genes regulated by constitutive promoter in the leaves of the source organ is higher than that in the ears of the pool organ and in rice (Jin et al. 2015). However, this did not affect the resistance of the transgenic lines to insect pests because the protein expression amplitude exceeded the lethal threshold of *C. suppressalis* (Ferré and Rie 2002; Chen et al. 2011). Finally, we found that the expression levels of *CRY* and *BAR* were high at heading stage, which might be related to the plant's own energy metabolism, and the vigorous capacity metabolism led to the high expression of exogenous genes (Ye et al. 2010).

Insect resistance was the first trait of interest in our study. The stem and young panicle of rice plants are the parts most susceptible to damage by *C. suppressalis*. We found that larval mortality was similar among varieties and years when they were exposed to transgenic stem tissue in the laboratory, and the resistance of all transgenic lines with the *CRY1C* or *CRY2A* gene to target pests was over 80%, which indicated that genetic background had little effect on the insect resistance of transgenic rice lines; these findings are consistent with the results of previous studies (Chen et al. 2005; Tang et al. 2006). In field trials under a pesticide-free environment, the percent dead hearts of the transgenic lines was below 2.5%, and the percent white spikelets was even lower than 1%; this indicates that the damage induced by insect pests was substantially inhibited. The slight variation in the percent dead hearts and white spikelets of non-transgenic control lines in the two years of the experiment indicated that non-transgenic control lines were affected by variation in the abundance of pests in these two years (Jiang et al. 2016).

Herbicide resistance was the second trait of interest in our study. The *EPSP* gene and *BAR* gene are the most widely used genes in herbicide-resistant transgenic crops (Green et al. 2014). The *BAR* gene from *Streptomyces hygroscopicus* used in this study confers high resistance to glyphosate herbicide. During the germination stage, Basta application had a stronger effect on shoot growth compared with root growth, and no effects of Basta application on transgenic lines were observed. Before the tillering stage, the chlorophyll content of transgenic lines began to return to normal after 2 d of herbicide application; at the tillering stage, the chlorophyll content of transgenic lines began to return to normal after 3 d of herbicide application. This difference is likely explained by differences in the energy demands associated with vegetative growth and reproductive growth. More substances and energy are used for the development of spikelets and floral organs during reproductive growth, and this reduces the expression of the *BAR* gene in the phloem of leaves and thus leads to a decrease in the chlorophyll content and photosynthetic efficiency. Basta began to be applied in the 1990s when transgenic crops were introduced, and previous studies have mainly focused on the effects of Basta on photosynthesis (Brookes et al. 2011). Previous studies have shown that Basta decreases the chlorophyll content and photosynthetic efficiency by impeding ammonia digestion in plants, which decreases ammonia accumulation. Generally, it takes 2 to 3 d for Basta to be transmitted to various parts of plants through the cuticle and cytoplasm (Vencill et al. 2012).

In our study, the yield of transgenic lines was significantly higher compared with non-transgenic lines in a pesticide-free environment. Analysis of yield components revealed that the percent dead hearts and percent white spikelets were low; thus, the number of undamaged panicles was high in transgenic lines because of their high resistance to insect pests and herbicides, and this high level of resistance was conferred by the *CRY1C* and *CRY2A* genes. In addition, few leaves in transgenic lines showed signs of damage, indicating that the introduction of the *BAR* gene and *CRY* gene promoted increases in the chlorophyll content and photosynthetic efficiency under exposure to insect pests and herbicides (Xiao et al. 2007; Wang et al. 2012). Non-targeted effects are also important to consider. In general, the introduction of foreign genes can have deleterious effects on yield traits (Jiang et al. 2014b; Liu et al. 2022). In field trials under normal pesticide management, no negative effects on important traits, such as the number of tillers, grains per panicle, and weight per 1,000 grain, were observed in all transgenic lines with the *CRY1C*, *CRY2A*, and *BAR* genes. This indicates that the yield traits of the transgenic lines were relatively stable compared with non-transgenic lines and that the transgenes had no substantial effect on yield traits.

## Methods

### Plant Materials

In this study, we used six *Bt*-transgenic rice lines, CHT025(1C), CHT025(2A), CH891(1C), CH891(2A), CH871(1C), and CH121(1C), and their non-transgenic counterparts: Changhui T025, Changhui 891, Changhui 871, and Changhui 121. Changhui T025, Changhui 891, Changhui 871, and Changhui 121 were backcross lines (Table 1). The seeds for these lines were cultivated by the Education Ministry Key Laboratory of Crop Physiology, Ecology, and Genetic Breeding at Jiangxi Agricultural University, Nanchang, China. *CRY1C*, *CRY2A* and *BAR* were transferred into the donor parent MH63 by Agrobacterium transformation. The pCAMBIA1300 was digested with XhoI, the hygromycin phosphotransferase (*hph*) gene was replaced with the phosphinotricin acetyltransferase (*BAR*) gene, flattened and then inserted into the bar gene to form an intermediate vector. The *BAR* gene was driven by CaMV 35S promoter (Cheng et al. 1998; Alam et al. 1999). Then the intermediate vector was double digested with HindIII + BamHI and connected to the ubiqultin promoter (Tang et al. 2006), After double digested with BamHI + SacI, inserted into the *CRY1C* and *CRY2A* became the final transformation vector. The *CRY1C* and *CRY2A* gene was driven by the rice ubiqultin promoter (Additional file 1: Fig S1). A strain of Agrobacterium tumefaciens EHA105, was used for the transformation experiments. The callus culture and transformation procedures were carried out as described by Hiei et al. (1994). The donor parent introduced the foreign gene into the recurrent parent, then backcross and marker-assisted selection were performed to obtain six *Bt*-transgenic rice lines.

**Table 1:** Bt-transgenic rice lines (BC_4_F_8_ and BC_4_F_9_) and their respective non-transgenic counterparts used for the experiments in 2020 and 2021

Type	Bt line	Control line
Inbred line	CHT025(1C)	Changhui T025
Inbred line	CHT025(2A)	Changhui T025
Inbred line	CH891(1C)	Changhui 891
Inbred line	CH891(2A)	Changhui 891
Inbred line	CH871(1C)	Changhui 871
Inbred line	CH121(1C)	Changhui 121

### Experimental Design

Field experiments were carried out from May to October in 2021 in the economic and technological development zone (28˚48′10″ N, 115˚49′55″ E) of Nanchang City, Jiangxi Province, China. Table 1 shows the mean monthly day and night temperature during the rice growing season. The six *Bt*-transgenic rice lines CHT025(1C), CHT025(2A), CH891(1C), CH891(2A), CH871(1C), and CH121(1C) and the control lines Changhui T025, Changhui 891, Changhui 871, and Changhui 121 were used in field experiments. The field experiment was conducted in a randomized block design with three replications. Each plot was 5 m × 5 m. Twenty-day-old seedlings were transplanted at a density of 15 cm × 20 cm with one seedling per hill. The soil type of the experimental site was reddish-yellow clay-like paddy soil. Soil in the upper 15 cm at the experimental site had the following properties at the start of the experiment: pH, 5.01; 1.26 g kg^–1^, total N; 105.6 mg kg^–1^, available phosphorus; 125.2 mg kg^–1^, potassium; and 20.56 g kg^−1^, organic matter. The experimental field was maintained in a flooded state from transplanting until 7 d before maturity. Standard management practices were applied to control the spread of pests and diseases and the growth of weeds to minimize yield losses.

### Genetic Background Detection

A total of 512 simple sequence repeat (SSR) primers spanning the whole rice genome were used to screen the genomes of three transgenic lines and their corresponding recurrent parents, and the genetic background recovery rate was determined. DNA was extracted using the cetyltrimethylammonium bromide (CTAB) method. The SSR primers were designed by the China Research Institute and synthesized by Shanghai Shenggong Biotechnology Company. The 15-μL PCR reactions included 1.5 µL of 10 × PCR buffer, 2 µL of 100 ng•μL^−1^ DNA template, 0.3 µL of 2.5 mmol• L^−1^ dNTPs, 10 µL of ddH_2_O, 0.5 µL of 10 μmol• L^−1^ positive and negative primers, and 0.2 µL of 5 U•μL^−1^ Taq DNA polymerase. The thermal cycling conditions were as follows: 94 °C for 5 min; 30 cycles of 94 °C for 1 min, 57 °C for 1 min, and 72 °C for 1 min; and 72 °C for 5 min. The PCR products were visualized using 8% polyacrylamide gel electrophoresis and rapid silver staining. The homozygous bands were labeled as 1, heterozygous bands were labeled as 2, and missing bands were labeled as 0.

### PCR and Southern Blot Analysis

Specific primers for *CRY1C*, *CRY2A* and *BAR* were designed for PCR reactions (Table 2). PCR was conducted using the procedure described in the previous section. The CTAB method was used to extract genomic DNA. For southern blot analysis, 5 µg of genomic DNA from each sample was digested with *Nco*I and *Hind*III, separated on a 0.8% agarose gel, and then transferred to a nylon membrane. A PCR-amplified fragment of *CRY1C* was used to prepare the probe. Next, 5 µg of genomic DNA from each sample was digested with *Bam*HI and *Eco*RI, separated on a 0.8% agarose gel, and then transferred to a nylon membrane. A PCR-amplified fragment of *CRY2A* was used to prepare the probe. Lastly, 5 µg of genomic DNA from each sample was digested with *Sam*I, followed by separation on a 0.8% agarose gel and then transfer to a nylon membrane. A PCR-amplified fragment of *BAR* was used to prepare the probe.

**Table 2:** PCR primers used in this study

Primer	Primer sequence	PCR product
*CRY1C*-F	TTCTACTGGGGAGGACATCG	799 bp
*CRY1C*-R	CGGTATCTTTGGGTGATTGG	
*CRY2A*-F	CGTGTCAATGCTGACCTGAT	600 bp
*CRY2A*-R	GATGCCGGACAGGATGTAGT	
*BAR*-F	GTCAACCACTACATCGAGACAAGC	460 bp
*BAR*-R	AGCAGGTGGGTGTAGAGCGT	

### Quantitative qRT-PCR Assays

Total RNA was extracted from tissues at various developmental stages by grinding in TRIzol (Merck, KGaA, Germany). DNase digestion was performed to avoid contamination from genomic DNA, and the phenol–chloroform method was used to isolate total RNA. The integrity of the extracted RNA was determined by 1.5% agarose gel electrophoresis, and RNA quantity and quality were measured using a NanoDrop 2000 spectrophotometer (Thermo Fisher Scientific Inc., Waltham, MA, USA) based on the 260/280-nm and 260/230-nm absorbance ratios. Complementary DNA was synthesized using a PrimeScript 1st Strand cDNA Synthesis Kit (TaKaRa, 6210A, Japan) according to the instructions of the PrimeScript RT Master Mix Kit. After ten-fold dilution of the cDNA, the target genes *CRY1C, CRY2A* and *BAR* and the reference gene Actin1 were detected by quantitative real-time PCR (qRT-PCR) according to the instructions of the SYBR Premix Taq II Kit (TaKaRa) (Table 3). The program of the 7500 Real-Time PCR system (Thermo Fisher Scientific) was incubation at 95 °C for 2 min, followed by 40 cycles of 95 °C for 15 s and 68 °C for 30 s, and a final extension step at 68 °C for 10 min. The purity of the amplicons was confirmed in the presence of a single peak in the melting curve (Agostinetto et al., 2019). Osactin1 was used as the internal reference gene for qRT-PCR normalization, and the qRT-PCR results were analyzed by the 2^−ΔΔCT^ method.

**Table 3:** qRT-PCR primers used in this study.

Primer	Primer sequence	Description
*CRY1C*-F	AAAGAATCGCTGAGTTCGCTAG	qRT-PCR
*CRY1C*-R	AAGAAGTCCATCAAGGATACGG	
*CRY2A*-F	TTCCTGCTGAAATAAGGTGGGT	qRT-PCR
*CRY2A*-R	ACGAGCGAGGGTGTCAGTGTT	
*BAR*-F	ACAAGCACGGTCAACTTCC	qRT-PCR
*BAR*-R	GAGGTCGTCCGTCCACTC	
*Actin*-F	TTCGGACCCAAGAATGCTAAG	qRT-PCR
*Actin*-R	AACAGATAGGCCGGTTGAAAAC	

### Quantitative ELISA Assay

The amount of Cry1C, Cry2A and Bar proteins in leaves, stems, and panicles was measured at the tillering, booting, heading, filling, and maturity stages using an ELISA kit (AP003 and AP005 CRBS; EnviroLogix Inc., Portland, ME, USA). The absorbance value was measured at 450 nm using a VICTOR Nivo multimode plate reader (PerkinElmer, Waltham, MA, USA). Based on the range of the standard curve, the Cry1C, Cry2A and Bar protein extract was diluted appropriately so that its absorbance value was within the range of the standard curve (Xu et al. 2018). For ELISA, a standard curve was drawn based on the absorbance of known concentrations of Cry1C, Cry2A and Bar standard (AP003 and AP005; EnviroLogix). The concentration of each test sample was determined from the standard curve, and the Cry1C, Cry2A and Bar protein content of the sample was calculated based on its dilution ratio and the conversion formula: Cry1C, Cry2A and Bar protein content (μg g^−1^ fresh weight) = test sample concentration (ng g^−1^) × dilution × extract volume/tissue fresh weight (mg).

### Insect Resistance in the Laboratory and the Field

Indoor insecticidal assays were conducted using 10 replicates of each transgenic line and non-transgenic control. An artificial diet was administered to *C. suppressalis* larvae for 9–10 d, and most of the borers developed to the second instar stage by 10 d. Second instar larvae were fed fresh transgenic rice leaves and stems that were collected from rice plants at the tillering and heading stages; non-transgenic rice tissues at the same growth stages were used as negative controls. Second instar larvae were placed on individual Petri dishes that contained a piece of leaf (4 g) or stem (5 g), and ddH_2_O was added to the filter paper to keep the environment of the Petri dish humid. Petri dishes were sealed with parafilm membranes to prevent larvae from escaping. All Petri dishes were stored in a hermetic box in the dark at approximately 27 ± 1˚C and 70 ± 10% relative humidity. The leaves were weighed after the in-situ assays were initiated; after 48 h, larval mortality was determined, and the mortality rate of *C. suppressalis* was calculated. The insect resistance of transgenic plants in the field was assessed by artificially infesting rice plants with *C. suppressalis*. Chemical insecticides that target lepidopteran pests were not applied throughout the experimental period. Field assays were conducted using three replicates of each transgenic line and non-transgenic control. Fifty individual plants were planted in each replicate. At the tillering stage, 15–20 first-instar *C. suppressalis* larvae were applied to each rice plant. The number of dead hearts induced by stem borers was counted at the end of the maximum tillering stage, and the number of white spikelets was counted at the flowering stage.

### Herbicide Resistance

The herbicide resistance of transgenic rice plants was evaluated at the bud stage through the application of the herbicide Basta. The seeds of six transgenic varieties and control varieties were germinated for 24 h, and ungerminated seeds were removed to ensure that the seed vigor would be 100% in the experiment. Treatments were conducted in a hydroponic system. The germinated seeds were placed into different hydroponic tanks and hydroponic solution per national standards. 10 mg/L Basta was added to the hydroponic solution, and plants were cultured at room temperature for 7 d, with the culture medium replaced every 3 d. Bud length and root length measurements were taken after the 7-d culture period.

Several varieties of genetically modified rice and non-genetically modified control seeds were germinated for one day, planted in pots, watered every three days, and fertilized on the tenth day after germination until the 27th d when 300 mg/L Basta was applied to the rice plants via foliar spraying. The chlorophyll content of rice plants was measured using a portable chlorophyll measurement instrument (SPAD, Soil and Plant Analyzer Development) at the seeding stage; chlorophyll content measurements were taken twice two days before Basta was applied, and once a day at 5:00 p.m. for 7 d following Basta application. The chlorophyll content in rice leaves was measured at the tillering stage, booting stage, and filling stage after non-transgenic rice plants were transplanted.

### Measurement of Grain Yield Traits

Transgenic rice plants were planted in a paddy field in the Transgenic Experimental Plots of Jiangxi Agricultural University (Nanchang, Jiangxi, China) for evaluation of agronomic performance. The non-transgenic control lines Changhui T025, Changhui 891, Changhui 871, and Changhui 121 were planted in paddy fields adjacent to the transgenic lines. Six randomly chosen blocks of 6 m^2^ (2 m × 3 m) were used in field trials. There were approximately 100 plants in each block, and each plant had approximately 10–15 tillers. The following five agronomic traits were measured for each plant: panicles per plant, grains per panicle, weight per 1,000 grains, and yield per plant.

### Data Analysis

After PCR bands were scored, the data were digitized in MS Excel, and linkage maps were constructed using CASS2.1 software. The recovery rate of the recurrent parent background was calculated using the formula E[G(g)] = 1-(1/2)^g+1^. The response rate of the genetic background was calculated using the formula G(g) = [L + X(g)]/2L, where G(g) indicates the response rate of the genetic background in the g generation, g indicates the number of generations used for backcrossing, L indicates the number of molecular markers involved in the analysis, and X(g) indicates the number of band markers in the backcross g generation that are the same as those of the recurrent parents. The gene and protein relative expression levels were processed and analyzed using Excel 2007 and SPSS 16.00 (IBM Corp., Armonk, NY, USA). The borer mortality rate data were processed and analyzed using Excel 2007 and SPSS 16.00. Agronomic traits of transgenic plants were compared with the recurrent parents using one-way analysis of variance (ANOVA). Values were presented as means (± SD). Data were analyzed by one-way ANOVA, and treatment means were compared using the least significant difference test at P = 0.05. Figures were constructed using Origin 2017 (OriginLab Corp., Northampton, MA, USA).

## Conclusion

The newly bred high-generation transgenic rice lines (BC_4_F_8_ and BC_4_F_9_) with *CRY1C*, *CRY2A*, and *BAR* genes all showed high genetic stability at the DNA, RNA, and protein levels. The high-generation transgenic lines also showed high resistance to insects and herbicides, and the yield of these transgenic lines was much higher than that of non-transgenic control lines in a pesticide-free environment. Under normal field management, the heterologous genes were stably inherited in these transgenic lines, and they had no effects on agronomic traits. Therefore, the resistance of the transgenic rice lines to insect pests and herbicides shows high genetic stability under various genetic backgrounds. Our findings indicate that *CRY1C*, *CRY2A*, and *BAR* could be used to breed new transgenic varieties.

## Supplementary Information

**Additional file 1： Table S1**. Genetic background response rate statistics. **Table S2**. Relative expression level of *CRY1C* and *CRY2A* in Bt-transgenic rice lines (BC_4_F_8_ and BC_4_F_9_). **Table S3**. Relative expression level of *CRY1C* and *CRY2A* in positive and negative control. **Table S4**. Relative expression level of *BAR* in Bt-transgenic rice lines (BC_4_F_8_ and BC_4_F_9_). **Table S5**. Relative expression level of BAR in positive and negative control. **Table S6**. Cry1C and Cry2A protein content (μg·g^−1^) in Bt-transgenic rice lines (BC_4_F_8_ and BC_4_F_9_). **Table S7**. Bar protein content (μg·g^−1^) in Bt-transgenic rice lines (BC_4_F_8_ and BC_4_F_9_). **Table S8**. Insect resistance of Bt-transgenic rice lines (BC_4_F_8_ and BC_4_F_9_) in the laboratory. **Table S9**. Insect resistance of Bt-transgenic rice lines (BC_4_F_8_ and BC_4_F_9_) in the field. **Table S10**. Herbicide resistance of Bt-transgenic rice lines (BC_4_F_8_ and BC_4_F_9_) in the laboratory. **Table S11**. Herbicide resistance of Bt-transgenic rice lines (BC_4_F_8_ and BC_4_F_9_) in the field. **Table S12**. Yields and relative traits of Bt-transgenic rice lines (BC_4_F_8_ and BC_4_F_9_) and their respective non-transgenic counterparts under pesticide-free environment. **Figure S1**. Construction of *CRY1C*, *CRY2A* and *BAR* vectors.

## Data Availability

The original contributions presented in the study are publicly available. The data sets supporting the results of this article are included within the article and its additional files. All experiment of plant and all field experiments are performed in our affiliated university.
