# Supplementary material for: Analysis of the Genetic Stability of Insect and Herbicide Resistance Genes in Transgenic Rice Lines: A Laboratory and Field Experiment
Source: Rice (N Y). 2023 Feb 13;16:8. doi: 10.1186/s12284-023-00624-5 (PMC9925649; doi:10.1186/s12284-023-00624-5)
Supplement: Supplementary file 1 — Additional file 1： Table S1. Genetic background response rate statistics. Table S2. Relative expression level of CRY1C and CRY2A in Bt-transgenic rice lines (BC4F8 and BC4F9). Table S3. Relative expression level of CRY1C and CRY2A in positive and negative control. Table S4. Relative expression level of BAR in Bt-transgenic rice lines (BC4F8 and BC4F9). Table S5. Relative expression level of BAR in positive and negative control. Table S6. Cry1C and Cry2A protein content (μg·g−1) in Bt-transgenic rice lines (BC4F8 and BC4F9). Table S7. Bar protein content (μg·g−1) in Bt-transgenic rice lines (BC4F8 and BC4F9). Table S8. Insect resistance of Bt-transgenic rice lines (BC4F8 and BC4F9) in the laboratory. Table S9. Insect resistance of Bt-transgenic rice lines (BC4F8 and BC4F9) in the field. Table S10. Herbicide resistance of Bt-transgenic rice lines (BC4F8 and BC4F9) in the laboratory. Table S11. Herbicide resistance of Bt-transgenic rice lines (BC4F8 and BC4F9) in the field. Table S12. Yields and relative traits of Bt-transgenic rice lines (BC4F8 and BC4F9) and their respective non-transgenic counterparts under pesticide-free environment. Figure S1. Construction of CRY1C, CRY2A and BAR vectors. [file 12284_2023_624_MOESM1_ESM.docx]

**Supporting Information**

**Table S1 Genetic background response rate statistics**

| Lines | Number of  SSR marker | Number of  polymorphic SSR  marker | Polymorphic  marker rate / % | Theoretical genetic  background response  rate / % | Actual genetic  background response  rate / % |
| --- | --- | --- | --- | --- | --- |
| CH121(1C) | 512 | 31 | 6.05 | 93. 75 | 96.97 |
| CH871(1C) | 512 | 10 | 1.95 | 93. 75 | 99.02 |
| CH891(1C) | 512 | 23 | 4.49 | 93. 75 | 97.75 |
| CH891(2A) | 512 | 11 | 2.15 | 93. 75 | 98.93 |
| CHT025(1C) | 512 | 21 | 4.10 | 93. 75 | 97.95 |
| CHT025(2A) | 512 | 25 | 4.88 | 93. 75 | 97.56 |

**Table S2 Relative expression level of *CRY1C* and *CRY2A* in Bt-transgenic rice lines (BC_4_F_8_ and BC_4_F_9_)**

| Year | Growth period | Tissue | Relative expression level of *CRY1C*/*CRY2A* (2^−ΔΔCT^) | | | | | |
| --- | --- | --- | --- | --- | --- | --- | --- | --- |
|  |  |  | CHT025(1C) | CHT025(2A) | CH891(1C) | CH891(2A) | CH871(1C) | CH121(1C) |
|  |  |  | *CRY1C* | *CRY2A* | *CRY1C* | *CRY2A* | *CRY1C* | *CRY1C* |
| 2020  BC_4_F_8_ | Seedling stage | Leaf | 6.08±0.30 | 0.59±0.02 | 0.29±0.21 | 1.16±0.14 | 1.22±0.15 | 1.55±0.50 |
|  | Seedling stage | Stem | 0.30±0.11 | 0.67±0.14 | 3.17±0.02 | 1.58±0.01 | 0.85±0.14 | 0.24±0.08 |
|  | Tillering stage | Leaf | 3.40±0.20 | 0.01±0.04 | 3.46±0.33 | 0.97±0.32 | 4.16±0.40 | 2.72±0.40 |
|  | Tillering stage | Stem | 2.56±0.12 | 4.30±0.21 | 0.47±0.11 | 2.38±0.12 | 0.84±0.21 | 0.19±0.10 |
|  | Booting stage | Leaf | 9.11±0.50 | 0.54±0.03 | 3.92±0.35 | 5.08±0.24 | 9.44±0.30 | 6.81±0.04 |
|  | Booting stage | Stem | 4.43±0.21 | 5.10±0.11 | 0.10±0.05 | 4.17±0.19 | 1.19±0.01 | 1.21±0.12 |
|  | Booting stage | Panicle | 0.10±0.01 | 0.62±0.03 | 1.99±0.02 | 0.07±0.02 | 0.41±0.01 | 0.17±0.02 |
|  | Heading stage | Leaf | 24.58±0.76 | 18.76±0.50 | 16.49±0.44 | 19.97±0.18 | 10.44±0.25 | 8.58±0.11 |
|  | Heading stage | Stem | 7.70±0.18 | 12.27±0.09 | 11.00±0.21 | 13.06±0.11 | 9.63±0.02 | 3.06±0.09 |
|  | Heading stage | Panicle | 2.94±0.06 | 1.86±0.02 | 3.05±0.03 | 4.59±0.01 | 1.80±0.02 | 1.16±0.03 |
|  | Maturation stage | Leaf | 8.86±0.24 | 0.06±0.02 | 2.48±0.42 | 3.38±0.36 | 1.01±0.20 | 5.73±0.04 |
|  | Maturation stage | Stem | 5.36±0.18 | 0.10±0.04 | 0.17±0.14 | 1.45±0.00 | 0.91±0.14 | 0.42±0.08 |
|  | Maturation stage | Panicle | 0.45±0.05 | 0.12±0.01 | 0.80±0.02 | 0.13±0.02 | 0.32±0.04 | 0.29±0.01 |
| 2021  BC_4_F_9_ | Seedling stage | Leaf | 9.01±0.15 | 0.43±0.03 | 0.29±0.14 | 2.11±0.26 | 1.50±0.20 | 0.47±0.04 |
|  | Seedling stage | Stem | 0.60±0.00 | 0.80±0.11 | 4.06±0.11 | 1.81±0.00 | 1.02±0.14 | 0.18±0.07 |
|  | Tillering stage | Leaf | 3.84±0.20 | 0.02±0.02 | 4.86±0.21 | 1.23±0.19 | 7.76±0.18 | 1.21±0.04 |
|  | Tillering stage | Stem | 4.36±0.15 | 6.73±0.18 | 0.48±0.12 | 2.19±0.14 | 0.77±0.15 | 0.29±0.06 |
|  | Booting stage | Leaf | 8.17±0.20 | 1.20±0.10 | 5.04±0.48 | 4.07±0.33 | 9.27±0.31 | 8.89±0.04 |
|  | Booting stage | Stem | 5.49±0.19 | 6.59±0.21 | 0.09±0.14 | 6.25±0.15 | 1.13±0.09 | 1.65±0.09 |
|  | Booting stage | Panicle | 0.06±0.04 | 1.07±0.02 | 2.19±0.01 | 0.07±0.01 | 0.33±0.02 | 0.13±0.01 |
|  | Heading stage | Leaf | 19.02±0.50 | 27.54±0.50 | 21.76±0.62 | 17.19±0.21 | 12.19±0.30 | 10.57±0.11 |
|  | Heading stage | Stem | 6.86±0.14 | 15.55±0.12 | 11.44±0.21 | 14.7±0.14 | 10.01±0.11 | 5.10±0.07 |
|  | Heading stage | Panicle | 2.77±0.02 | 3.41±0.02 | 2.55±0.02 | 5.43±0.02 | 1.54±0.03 | 3.67±0.03 |
|  | Maturation stage | Leaf | 7.03±0.60 | 0.04±0.03 | 3.31±0.36 | 3.26±0.35 | 1.01±0.45 | 5.43±0.04 |
|  | Maturation stage | Stem | 4.87±0.21 | 0.20±0.09 | 0.23±0.09 | 1.38±0.00 | 0.90±0.08 | 1.62±0.05 |
|  | Maturation stage | Panicle | 0.41±0.03 | 0.53±0.01 | 0.69±0.02 | 0.17±0.02 | 0.24±0.02 | 0.29±0.01 |

Note: The data presented are the mean ± standard deviation, n = 3.

**Table S3 Relative expression level of *CRY1C* and *CRY2A* in positive and negative control**

| generation | Growth period | Tissue | Relative expression level of *CRY1C*/*CRY2A* (2^−ΔΔCT^) | | | | | | | | | | | |
| --- | --- | --- | --- | --- | --- | --- | --- | --- | --- | --- | --- | --- | --- | --- |
|  |  |  | MH63(1C)  CHT025(1C)Positive control | CHT025  CHT025(1C) negative control | MH63(2A)  CHT025(2A)Positive control | CHT025  CHT025(2A)negative control | MH63(1C)  CH891(1C )Positive control | CH891  CH891(1C) negative control | MH63(2A)  CH891(2A) Positive control | CH891  CH891(2A) negative control | MH63(1C)  CH871(1C )Positive control | CH871  CH871(1C) negative control | MH63(1C)  CH121(1C )Positive control | CH121  CH121(1C) negative control |
|  |  |  | *CRY1C* | *CRY1C* | *CRY2A* | *CRY2A* | *CRY1C* | *CRY1C* | *CRY2A* | *CRY2A* | *CRY1C* | *CRY1C* | *CRY1C* | *CRY1C* |
| 2020 | Seedling stage | Leaf | 1.00±0.20 | 0.00±0.00 | 1.50±0.30 | 0.00±0.00 | 1.00±0.20 | 0.00±0.00 | 1.50±0.30 | 0.00±0.00 | 1.00±0.20 | 0.00±0.00 | 1.00±0.20 | 0.00±0.00 |
|  | Seedling stage | Stem | 0.87±0.20 | 0.00±0.00 | 1.23±0.15 | 0.00±0.00 | 0.87±0.20 | 0.00±0.00 | 1.23±0.15 | 0.00±0.00 | 0.87±0.20 | 0.00±0.00 | 0.87±0.20 | 0.00±0.00 |
|  | Tillering stage | Leaf | 5.28±0.30 | 0.00±0.00 | 4.30±0.40 | 0.00±0.00 | 5.28±0.30 | 0.00±0.00 | 4.30±0.40 | 0.00±0.00 | 5.28±0.30 | 0.00±0.00 | 5.28±0.30 | 0.00±0.00 |
|  | Tillering stage | Stem | 1.59±0.15 | 0.00±0.00 | 2.45±0.18 | 0.00±0.00 | 1.59±0.15 | 0.00±0.00 | 2.45±0.18 | 0.00±0.00 | 1.59±0.15 | 0.00±0.00 | 1.59±0.15 | 0.00±0.00 |
|  | Booting  stage | Leaf | 12.74±0.60 | 0.00±0.00 | 6.90±0.50 | 0.00±0.00 | 12.74±0.60 | 0.00±0.00 | 6.90±0.50 | 0.00±0.00 | 12.74±0.60 | 0.00±0.00 | 12.74±0.60 | 0.00±0.00 |
|  | Booting  stage | Stem | 3.42±0.20 | 0.00±0.00 | 4.89±0.22 | 0.00±0.00 | 3.42±0.20 | 0.00±0.00 | 4.89±0.22 | 0.00±0.00 | 3.42±0.20 | 0.00±0.00 | 3.42±0.20 | 0.00±0.00 |
|  | Booting  stage | Panicle | 0.20±0.05 | 0.00±0.00 | 0.18±0.03 | 0.00±0.00 | 0.20±0.05 | 0.00±0.00 | 0.18±0.03 | 0.00±0.00 | 0.20±0.05 | 0.00±0.00 | 0.20±0.05 | 0.00±0.00 |
|  | Heading stage | Leaf | 16.42±0.50 | 0.00±0.00 | 12.40±0.60 | 0.00±0.00 | 16.42±0.50 | 0.00±0.00 | 12.40±0.60 | 0.00±0.00 | 16.42±0.50 | 0.00±0.00 | 16.42±0.50 | 0.00±0.00 |
|  | Heading stage | Stem | 6.68±0.12 | 0.00±0.00 | 8.54±0.15 | 0.00±0.00 | 6.68±0.12 | 0.00±0.00 | 8.54±0.15 | 0.00±0.00 | 6.68±0.12 | 0.00±0.00 | 6.68±0.12 | 0.00±0.00 |
|  | Heading stage | Panicle | 2.30±0.08 | 0.00±0.00 | 3.40±0.05 | 0.00±0.00 | 2.30±0.08 | 0.00±0.00 | 3.40±0.05 | 0.00±0.00 | 2.30±0.08 | 0.00±0.00 | 2.30±0.08 | 0.00±0.00 |
|  | Maturation stage | Leaf | 4.52±0.40 | 0.00±0.00 | 4.30±0.40 | 0.00±0.00 | 4.52±0.40 | 0.00±0.00 | 4.30±0.40 | 0.00±0.00 | 4.52±0.40 | 0.00±0.00 | 4.52±0.40 | 0.00±0.00 |
|  | Maturation stage | Stem | 4.35±0.11 | 0.00±0.00 | 3.21±0.18 | 0.00±0.00 | 4.35±0.11 | 0.00±0.00 | 3.21±0.18 | 0.00±0.00 | 4.35±0.11 | 0.00±0.00 | 4.35±0.11 | 0.00±0.00 |
|  | Maturation stage | Panicle | 0.50±0.06 | 0.00±0.00 | 0.74±0.02 | 0.00±0.00 | 0.50±0.06 | 0.00±0.00 | 0.74±0.02 | 0.00±0.00 | 0.50±0.06 | 0.00±0.00 | 0.50±0.06 | 0.00±0.00 |

Note: The data presented are the mean ± standard deviation, n = 3.

**Table S4 Relative expression level of *BAR* in Bt-transgenic rice lines (BC_4_F_8_ and BC_4_F_9_)**

| Year | Growth period | Tissue | Relative expression level of *BAR* (2^−ΔΔCT^) | | | | | |
| --- | --- | --- | --- | --- | --- | --- | --- | --- |
|  |  |  | CHT025(1C) | CHT025(2A) | CH891(1C) | CH891(2A) | CH871(1C) | CH121(2A) |
|  |  |  | *BAR* | *BAR* | *BAR* | *BAR* | *BAR* | *BAR* |
| 2020  BC_4_F_8_ | Seedling stage | Leaf | 0.86±0.10 | 1.92±0.10 | 0.18±0.02 | 1.00±0.40 | 1.20±0.20 | 4.88±0.40 |
|  | Seedling stage | Stem | 0.19±0.01 | 0.25±0.10 | 1.28±0.20 | 0.85±0.40 | 2.15±0.20 | 0.13±0.40 |
|  | Tillering stage | Leaf | 0.00±0.00 | 0.00±0.00 | 3.87±0.40 | 0.29±0.01 | 0.68±0.30 | 2.53±0.50 |
|  | Tillering stage | Stem | 0.05±0.00 | 1.94±0.40 | 1.69±0.40 | 2.98±0.01 | 2.35±0.30 | 0.22±0.50 |
|  | Booting stage | Leaf | 9.63±0.20 | 7.14±0.20 | 0.42±0.02 | 3.00±0.40 | 1.96±0.10 | 1.42±0.10 |
|  | Booting stage | Stem | 1.76±0.20 | 6.44±0.20 | 0.63±0.02 | 0.51±0.40 | 0.67±0.10 | 7.75±0.10 |
|  | Booting stage | Panicle | 1.76±0.20 | 6.44±0.20 | 0.07±0.02 | 0.51±0.02 | 0.67±0.10 | 6.44±0.10 |
|  | Heading stage | Leaf | 28.21±0.40 | 35.47±0.40 | 6.19±0.50 | 9.82±0.80 | 18.92±0.50 | 8.68±0.80 |
|  | Heading stage | Stem | 5.41±0.40 | 4.24±0.40 | 3.53±0.50 | 6.42±0.08 | 1.16±0.50 | 2.77±0.80 |
|  | Heading stage | Panicle | 5.41±0.40 | 4.24±0.40 | 4.53±0.50 | 6.43±0.50 | 1.16±0.50 | 4.24±0.80 |
|  | Maturation stage | Leaf | 6.07±0.40 | 5.44±0.40 | 0.86±0.20 | 1.28±0.30 | 0.02±0.00 | 1.62±0.40 |
|  | Maturation stage | Stem | 2.19±0.40 | 2.98±0.40 | 0.30±0.02 | 2.67±0.30 | 1.40±0.30 | 4.42±0.40 |
|  | Maturation stage | Panicle | 2.19±0.40 | 2.98±0.40 | 0.96±0.20 | 2.68±0.20 | 1.40±0.30 | 2.98±0.40 |
| 2021  BC_4_F_9_ | Seedling stage | Leaf | 1.80±0.10 | 1.56±0.10 | 0.47±0.03 | 1.80±0.30 | 2.83±0.10 | 3.73±0.40 |
|  | Seedling stage | Stem | 0.29±0.01 | 0.21±0.01 | 1.29±0.03 | 1.53±0.30 | 1.52±0.10 | 0.10±0.04 |
|  | Tillering stage | Leaf | 0.00±0.00 | 0.00±0.00 | 6.11±0.50 | 0.28±0.01 | 1.42±0.50 | 2.47±0.50 |
|  | Tillering stage | Stem | 0.04±0.01 | 1.72±0.02 | 1.37±0.50 | 3.93±0.01 | 4.42±0.50 | 0.21±0.05 |
|  | Booting stage | Leaf | 6.80±0.20 | 7.55±0.20 | 0.29±0.06 | 2.15±0.30 | 4.70±0.10 | 2.36±0.10 |
|  | Booting stage | Stem | 1.46±0.10 | 8.54±0.20 | 0.58±0.06 | 0.75±0.30 | 1.59±0.10 | 2.26±0.10 |
|  | Booting stage | Panicle | 1.46±0.10 | 8.54±0.10 | 0.08±0.06 | 0.75±0.06 | 1.59±0.10 | 8.54±0.10 |
|  | Heading stage | Leaf | 22.74±0.40 | 32.67±0.40 | 10.14±0.70 | 7.66±0.90 | 18.23±0.20 | 9.45±0.80 |
|  | Heading stage | Stem | 4.05±0.30 | 3.48±0.40 | 2.82±0.70 | 6.31±0.90 | 1.54±0.20 | 1.77±0.80 |
|  | Heading stage | Panicle | 4.05±0.30 | 3.48±0.30 | 4.54±0.70 | 6.30±0.70 | 1.54±0.20 | 3.48±0.80 |
|  | Maturation stage | Leaf | 3.58±0.40 | 3.53±0.40 | 1.41±0.40 | 0.94±0.40 | 0.04±0.00 | 2.25±0.40 |
|  | Maturation stage | Stem | 3.38±0.20 | 2.99±0.40 | 0.25±0.04 | 1.11±0.11 | 1.01±0.00 | 4.24±0.04 |
|  | Maturation stage | Panicle | 3.38±0.20 | 2.99±0.20 | 0.58±0.40 | 1.11±0.40 | 1.01±0.00 | 2.99±0.40 |

Note: The data presented are the mean ± standard deviation, n = 3.

**Table S5 Relative expression level of *BAR* in positive and negative control**

| generation | Growth period | Tissue | Relative expression level of *BAR* (2^−ΔΔCT^) | | | | | | | | | | | |
| --- | --- | --- | --- | --- | --- | --- | --- | --- | --- | --- | --- | --- | --- | --- |
|  |  |  | MH63(1C)  CHT025(1C)Positive control | CHT025  CHT025(1C) negative control | MH63(2A)  CHT025(2A)Positive control | CHT025  CHT025(2A)negative control | MH63(1C)  CH891(1C )Positive control | CH891  CH891(1C) negative control | MH63(2A)  CH891(2A) Positive control | CH891  CH891(2A) negative control | MH63(1C)  CH871(1C )Positive control | CH871  CH871(1C) negative control | MH63(1C)  CH121(1C )Positive control | CH121  CH121(1C) negative control |
|  |  |  | *BAR* | *BAR* | *BAR* | *BAR* | *BAR* | *BAR* | *BAR* | *BAR* | *BAR* | *BAR* | *BAR* | *BAR* |
| 2020 | Seedling stage | Leaf | 4.00±0.30 | 0.00±0.00 | 4.00±0.30 | 0.00±0.00 | 4.00±0.30 | 0.00±0.00 | 4.00±0.30 | 0.00±0.00 | 4.00±0.30 | 0.00±0.00 | 4.00±0.30 | 0.00±0.00 |
|  | Seedling stage | Stem | 1.25±0.30 | 0.00±0.00 | 1.25±0.30 | 0.00±0.00 | 1.25±0.30 | 0.00±0.00 | 1.25±0.30 | 0.00±0.00 | 1.25±0.30 | 0.00±0.00 | 1.25±0.30 | 0.00±0.00 |
|  | Tillering stage | Leaf | 2.15±0.20 | 0.00±0.00 | 2.15±0.20 | 0.00±0.00 | 2.15±0.20 | 0.00±0.00 | 2.15±0.20 | 0.00±0.00 | 2.15±0.20 | 0.00±0.00 | 2.15±0.20 | 0.00±0.00 |
|  | Tillering stage | Stem | 2.47±0.20 | 0.00±0.00 | 2.47±0.20 | 0.00±0.00 | 2.47±0.20 | 0.00±0.00 | 2.47±0.20 | 0.00±0.00 | 2.47±0.20 | 0.00±0.00 | 2.47±0.20 | 0.00±0.00 |
|  | Booting stage | Leaf | 8.00±0.40 | 0.00±0.00 | 8.00±0.40 | 0.00±0.00 | 8.00±0.40 | 0.00±0.00 | 8.00±0.40 | 0.00±0.00 | 8.00±0.40 | 0.00±0.00 | 8.00±0.40 | 0.00±0.00 |
|  | Booting stage | Stem | 1.52±0.40 | 0.00±0.00 | 1.52±0.40 | 0.00±0.00 | 1.52±0.40 | 0.00±0.00 | 1.52±0.40 | 0.00±0.00 | 1.52±0.40 | 0.00±0.00 | 1.52±0.40 | 0.00±0.00 |
|  | Booting stage | Panicle | 7.42±0.40 | 0.00±0.00 | 7.42±0.40 | 0.00±0.00 | 7.42±0.40 | 0.00±0.00 | 7.42±0.40 | 0.00±0.00 | 7.42±0.40 | 0.00±0.00 | 7.42±0.40 | 0.00±0.00 |
|  | Heading stage | Leaf | 21.00±0.50 | 0.00±0.00 | 21.00±0.50 | 0.00±0.00 | 21.00±0.50 | 0.00±0.00 | 21.00±0.50 | 0.00±0.00 | 21.00±0.50 | 0.00±0.00 | 21.00±0.50 | 0.00±0.00 |
|  | Heading stage | Stem | 4.34±0.50 | 0.00±0.00 | 4.34±0.50 | 0.00±0.00 | 4.34±0.50 | 0.00±0.00 | 4.34±0.50 | 0.00±0.00 | 4.34±0.50 | 0.00±0.00 | 4.34±0.50 | 0.00±0.00 |
|  | Heading stage | Panicle | 3.21±0.50 | 0.00±0.00 | 3.21±0.50 | 0.00±0.00 | 3.21±0.50 | 0.00±0.00 | 3.21±0.50 | 0.00±0.00 | 3.21±0.50 | 0.00±0.00 | 3.21±0.50 | 0.00±0.00 |
|  | Maturation stage | Leaf | 3.00±0.20 | 0.00±0.00 | 3.00±0.20 | 0.00±0.00 | 3.00±0.20 | 0.00±0.00 | 3.00±0.20 | 0.00±0.00 | 3.00±0.20 | 0.00±0.00 | 3.00±0.20 | 0.00±0.00 |
|  | Maturation stage | Stem | 1.68±0.20 | 0.00±0.00 | 1.68±0.20 | 0.00±0.00 | 1.68±0.20 | 0.00±0.00 | 1.68±0.20 | 0.00±0.00 | 1.68±0.20 | 0.00±0.00 | 1.68±0.20 | 0.00±0.00 |
|  | Maturation stage | Panicle | 1.97±0.20 | 0.00±0.00 | 1.97±0.20 | 0.00±0.00 | 1.97±0.20 | 0.00±0.00 | 1.97±0.20 | 0.00±0.00 | 1.97±0.20 | 0.00±0.00 | 1.97±0.20 | 0.00±0.00 |

Note: The data presented are the mean ± standard deviation, n = 3.

**Table S6 Cry1C and Cry2A protein content (μg·g^−1^) in Bt-transgenic rice lines (BC_4_F_8_ and BC_4_F_9_)**

| Year | Growth period | Tissue | Cry1C/Cry2A protein content (μg·g^−1^) | | | | | |
| --- | --- | --- | --- | --- | --- | --- | --- | --- |
|  |  |  | CHT025(1C) | CHT025(2A) | CH891(1C) | CH891(2A) | CH871(1C) | CH121(2A) |
|  |  |  | Cry1C | Cry2A | Cry1C | Cry2A | Cry1C | Cry1C |
| 2020  BC_4_F_8_ | Seedling stage | Leaf | 5.71±0.20 | 35.14±2.20 | 10.20±0.21 | 42.61±2.20 | 5.08±0.20 | 3.96±0.20 |
|  | Seedling stage | Stem | 3.90±0.11 | 33.56±1.40 | 0.40±0.02 | 10.56±0.01 | 3.08±0.20 | 2.74±0.08 |
|  | Tillering stage | Leaf | 4.70±0.10 | 42.84±2.10 | 10.05±0.33 | 57.09±3.10 | 4.69±0.10 | 4.23±0.10 |
|  | Tillering stage | Stem | 3.71±0.12 | 38.92±1.30 | 2.73±0.11 | 11.36±0.12 | 4.82±0.10 | 3.48±0.10 |
|  | Booting stage | Leaf | 5.27±0.50 | 50.43±1.20 | 7.23±0.35 | 72.96±4.20 | 3.35±0.50 | 5.46±0.50 |
|  | Booting stage | Stem | 4.07±0.21 | 34.04±1.10 | 7.23±0.05 | 23.15±0.19 | 3.47±0.50 | 2.16±0.12 |
|  | Booting stage | Panicle | 2.23±0.08 | 14.25±1.08 | 1.42±0.08 | 18.25±2.08 | 1.70±0.01 | 1.70±0.12 |
|  | Heading stage | Leaf | 7.16±0.40 | 50.43±2.40 | 16.16±0.44 | 73.33±3.40 | 7.22±0.40 | 15.64±0.40 |
|  | Heading stage | Stem | 4.96±0.18 | 41.96±1.80 | 2.87±0.21 | 12.64±0.11 | 4.83±0.40 | 3.82±0.09 |
|  | Heading stage | Panicle | 3.98±0.12 | 21.74±2.32 | 3.44±0.12 | 39.42±3.12 | 2.58±0.02 | 2.58±0.09 |
|  | Maturation stage | Leaf | 4.20±0.30 | 34.39±1.30 | 16.48±0.42 | 50.78±2.30 | 5.20±0.30 | 6.90±0.30 |
|  | Maturation stage | Stem | 3.58±0.18 | 37.25±1.90 | 1.86±0.14 | 9.96±0.11 | 4.43±0.30 | 3.40±0.08 |
|  | Maturation stage | Panicle | 1.76±0.01 | 8.42±0.51 | 0.29±0.01 | 2.02±0.21 | 0.76±0.04 | 0.76±0.08 |
| 2021  BC_4_F_9_ | Seedling stage | Leaf | 4.82±0.20 | 34.78±1.20 | 10.86±0.14 | 40.25±1.50 | 4.81±0.20 | 3.45±0.20 |
|  | Seedling stage | Stem | 3.91±0.21 | 32.97±1.20 | 1.84±0.11 | 8.26±0.11 | 3.81±0.20 | 2.19±0.07 |
|  | Tillering stage | Leaf | 4.12±0.30 | 42.97±1.30 | 8.73±0.21 | 48.78±1.80 | 3.61±0.30 | 3.68±0.30 |
|  | Tillering stage | Stem | 4.14±0.15 | 35.83±1.30 | 3.75±0.12 | 16.19±0.14 | 3.48±0.30 | 4.04±0.06 |
|  | Booting stage | Leaf | 4.74±0.30 | 45.59±1.30 | 10.24±0.48 | 50.74±2.30 | 3.21±0.30 | 6.59±0.30 |
|  | Booting stage | Stem | 4.22±0.19 | 37.79±1.10 | 3.42±0.14 | 25.48±0.15 | 4.16±0.30 | 3.38±0.09 |
|  | Booting stage | Panicle | 2.01±0.12 | 20.74±1.12 | 2.39±0.12 | 12.31±1.12 | 1.20±0.02 | 1.20±0.09 |
|  | Heading stage | Leaf | 9.28±0.50 | 55.59±2.50 | 12.19±0.62 | 59.61±2.50 | 7.62±0.50 | 10.74±0.50 |
|  | Heading stage | Stem | 4.25±0.14 | 44.23±1.50 | 4.38±0.21 | 14.89±0.14 | 6.21±0.50 | 5.09±0.07 |
|  | Heading stage | Panicle | 3.79±0.13 | 24.32±1.63 | 4.12±0.13 | 30.15±2.13 | 2.37±0.03 | 2.37±0.07 |
|  | Maturation stage | Leaf | 3.53±0.20 | 34.82±1.20 | 16.42±0.36 | 44.78±1.60 | 4.38±0.20 | 4.38±0.20 |
|  | Maturation stage | Stem | 3.90±0.21 | 35.65±1.20 | 3.15±0.09 | 8.11±0.11 | 3.40±0.20 | 1.85±0.05 |
|  | Maturation stage | Panicle | 2.49±0.02 | 6.31±0.42 | 0.76±0.02 | 1.65±0.22 | 0.49±0.02 | 0.49±0.05 |

Note: The data presented are the mean ± standard deviation, n = 3.

**Table S7 Bar protein content (μg·g^−1^) in Bt-transgenic rice lines (BC_4_F_8_ and BC_4_F_9_)**

| Year | Growth period | Tissue | Bar protein content (μg·g^−1^) | | | | | |
| --- | --- | --- | --- | --- | --- | --- | --- | --- |
|  |  |  | CHT025(1C) | CHT025(2A) | CH891(1C) | CH891(2A) | CH871(1C) | CH121(2A) |
|  |  |  | Bar | Bar | Bar | Bar | Bar | Bar |
| 2020  BC_4_F_8_ | Seedling stage | Leaf | 1.81±0.02 | 2.15±0.02 | 1.82±0.11 | 1.75±0.04 | 1.48±0.12 | 3.15±0.20 |
|  | Seedling stage | Stem | 1.04±0.06 | 1.74±0.14 | 1.20±0.02 | 1.64±0.07 | 1.42±0.12 | 2.16±0.08 |
|  | Tillering stage | Leaf | 1.36±0.04 | 3.48±0.21 | 1.35±0.13 | 2.18±0.01 | 1.87±0.11 | 4.16±0.10 |
|  | Tillering stage | Stem | 0.63±0.02 | 1.68±0.13 | 0.73±0.11 | 1.74±0.12 | 1.65±0.11 | 1.98±0.10 |
|  | Booting stage | Leaf | 2.08±0.05 | 3.79±0.21 | 2.00±0.15 | 2.58±0.14 | 1.76±0.15 | 4.72±0.50 |
|  | Booting stage | Stem | 0.33±0.01 | 1.21±0.11 | 1.03±0.05 | 1.14±0.19 | 1.21±0.15 | 2.19±0.12 |
|  | Booting stage | Panicle | 0.74±0.08 | 0.96±0.08 | 1.10±0.08 | 1.12±0.08 | 1.87±0.08 | 1.24±0.05 |
|  | Heading stage | Leaf | 2.19±0.14 | 4.16±0.40 | 2.62±0.14 | 3.48±0.28 | 2.00±0.14 | 5.21±0.40 |
|  | Heading stage | Stem | 1.73±0.18 | 2.14±0.14 | 1.83±0.21 | 1.87±0.11 | 1.74±0.14 | 2.69±0.09 |
|  | Heading stage | Panicle | 1.51±0.12 | 1.21±0.12 | 1.98±0.12 | 1.66±0.12 | 2.15±0.12 | 1.74±0.09 |
|  | Maturation stage | Leaf | 0.49±0.03 | 1.04±0.03 | 1.15±0.12 | 0.76±0.03 | 0.81±0.03 | 1.46±0.30 |
|  | Maturation stage | Stem | 0.03±0.01 | 0.34±0.02 | 0.22±0.04 | 0.32±0.01 | 0.34±0.13 | 0.92±0.08 |
|  | Maturation stage | Panicle | 0.30±0.01 | 0.15±0.01 | 0.38±0.01 | 0.15±0.01 | 0.30±0.04 | 1.44±0.08 |
| 2021  BC_4_F_9_ | Seedling stage | Leaf | 1.73±0.02 | 2.67±0.12 | 1.73±0.14 | 1.65±0.03 | 1.48±0.04 | 4.38±0.20 |
|  | Seedling stage | Stem | 1.17±0.03 | 1.21±0.12 | 1.51±0.11 | 1.58±0.11 | 1.94±0.12 | 1.89±0.07 |
|  | Tillering stage | Leaf | 2.41±0.03 | 4.15±0.23 | 1.67±0.11 | 2.47±0.01 | 2.15±0.13 | 5.19±0.30 |
|  | Tillering stage | Stem | 1.49±0.05 | 1.68±0.13 | 1.58±0.12 | 1.65±0.14 | 2.15±0.13 | 2.16±0.06 |
|  | Booting stage | Leaf | 2.56±0.03 | 3.89±0.23 | 1.61±0.18 | 2.49±0.13 | 2.05±0.13 | 3.72±0.30 |
|  | Booting stage | Stem | 1.11±0.02 | 1.83±0.18 | 2.58±0.14 | 1.89±0.15 | 1.14±0.13 | 2.14±0.09 |
|  | Booting stage | Panicle | 0.96±0.12 | 1.42±0.12 | 1.20±0.12 | 0.74±0.02 | 1.96±0.09 | 1.35±0.09 |
|  | Heading stage | Leaf | 2.57±0.15 | 4.15±0.50 | 1.92±0.12 | 3.48±0.39 | 2.15±0.15 | 6.58±0.50 |
|  | Heading stage | Stem | 1.86±0.14 | 2.14±0.12 | 3.42±0.21 | 1.97±0.14 | 2.94±0.15 | 2.49±0.07 |
|  | Heading stage | Panicle | 1.70±0.13 | 1.96±0.13 | 1.64±0.13 | 0.89±0.03 | 2.25±0.13 | 1.72±0.07 |
|  | Maturation stage | Leaf | 0.80±0.02 | 0.76±0.02 | 1.24±0.16 | 1.03±0.04 | 0.81±0.02 | 0.99±0.20 |
|  | Maturation stage | Stem | 0.34±0.02 | 0.48±0.03 | 0.12±0.03 | 0.56±0.02 | 0.34±0.12 | 0.51±0.05 |
|  | Maturation stage | Panicle | 0.71±0.02 | 0.21±0.02 | 0.49±0.02 | 0.09±0.02 | 0.30±0.02 | 0.32±0.05 |

Note: The data presented are the mean ± standard deviation, n = 3.

**Table S8 Insect resistance of Bt-transgenic rice lines (BC_4_F_8_ and BC_4_F_9_) in the laboratory**

| Year | Larval mortality (%) | | | | | | | | | |
| --- | --- | --- | --- | --- | --- | --- | --- | --- | --- | --- |
|  | CHT025(1C) | CHT025(2A) | CH891(1C) | CH891(2A) | CH871(1C) | CH121(1C) | CHT025 | CH891 | CH871 | CH121 |
| 2020  BC_4_F_8_ | 80.00±9.69 | 77.78±13.33 | 84.44±3.85 | 80.00±3.85 | 88.89±7.37 | 71.11±7.37 | 25.00±5.28 | 22.73±1.28 | 24.44±3.14 | 20.86±0.85 |
| 2021  BC_4_F_9_ | 97.06±2.16 | 94.12±4.32 | 84.44±13.15 | 82.22±6.29 | 87.06±4.16 | 93.90±4.32 | 28.70±1.25 | 27.53±2.17 | 23.17±1.86 | 30.50±6.41 |

Note: The data presented are the mean ± standard deviation, n = 10.

**Table S9 Insect resistance of Bt-transgenic rice lines (BC_4_F_8_ and BC_4_F_9_) in the field**

| Year | Lines | Stem at tillering stage | Stem at heading stage |
| --- | --- | --- | --- |
|  |  | Rate(%) of deadhearts | Rate(%) of white spikelets |
| 2020  BC_4_F_8_ | CHT025(1C) | 0.71±0.23 | 0.31±0.14 |
|  | CHT025(2A) | 0.31±0.14 | 0.15±0.10 |
|  | CH891(1C) | 0.71±0.23 | 0.63±0.21 |
|  | CH891(2A) | 0.31±0.14 | 0.31±0.14 |
|  | CH871(1C) | 0.72±0.24 | 0.71±0.23 |
|  | CH121(1C) | 0.71±0.23 | 0.03±0.01 |
|  | CHT025 | 22.86±1.74 | 28.70±1.25 |
|  | CHT025 | 21.30±1.56 | 27.53±2.17 |
|  | CH891 | 22.83±0.82 | 34.10±2.62 |
|  | CH891 | 20.86±0.85 | 31.30±3.41 |
|  | CH871 | 22.25±1.15 | 28.17±1.86 |
|  | CH121 | 22.73±1.28 | 32.30±2.69 |
| 2021  BC_4_F_9_ | CHT025(1C) | 0.31±0.14 | 0.55±0.14 |
|  | CHT025(2A) | 0.71±0.23 | 0.63±0.12 |
|  | CH891(1C) | 0.71±0.23 | 0.63±0.15 |
|  | CH891(2A) | 1.36±0.17 | 0.31±0.04 |
|  | CH871(1C) | 1.91±0.45 | 0.46±0.09 |
|  | CH121(1C) | 2.14±0.57 | 0.66±0.12 |
|  | CHT025 | 17.03±2.97 | 33.00±1.28 |
|  | CHT025 | 17.96±1.64 | 32.30±2.69 |
|  | CH891 | 18.31±1.79 | 33.40±3.26 |
|  | CH891 | 19.51±2.29 | 28.70±1.25 |
|  | CH871 | 18.20±2.40 | 27.53±2.17 |
|  | CH121 | 18.83±1.47 | 33.17±1.86 |

Note: The data presented are the mean ± standard deviation, n = 3.

**Table S10 Herbicide resistance of Bt-transgenic rice lines (BC_4_F_8_ and BC_4_F_9_) in the laboratory**

| Year | Lines | Bud length (cm) | Root length (cm) |
| --- | --- | --- | --- |
| 2020  BC_4_F_8_ | CHT025(1C) | 4.25±0.49 | 1.03±0.18 |
|  | CHT025(2A) | 3.85±0.57 | 0.98±0.33 |
|  | CH891(1C) | 3.04±0.73 | 0.89±0.25 |
|  | CH891(2A) | 4.10±0.32 | 1.08±0.40 |
|  | CH871(1C) | 3.38±0.85 | 0.73±0.34 |
|  | CH121(1C) | 4.28±0.26 | 1.08±0.40 |
|  | CHT025 | 0.95±0.21 | 0.43±0.17 |
|  | CH891 | 0.50±0.32 | 0.35±0.24 |
|  | CH871 | 0.83±0.10 | 0.45±0.24 |
|  | CH121 | 0.75±0.34 | 0.38±0.28 |
| 2021  BC_4_F_9_ | CHT025(1C) | 4.30±0.57 | 1.03±0.13 |
|  | CHT025(2A) | 4.05±0.79 | 1.03±0.14 |
|  | CH891(1C) | 4.06±0.24 | 1.08±0.11 |
|  | CH891(2A) | 3.39±0.30 | 0.85±0.14 |
|  | CH871(1C) | 4.08±0.56 | 1.05±0.12 |
|  | CH121(1C) | 4.08±0.67 | 0.80±0.18 |
|  | CHT025 | 1.00±0.28 | 0.58±0.22 |
|  | CH891 | 0.68±0.36 | 0.40±0.15 |
|  | CH871 | 0.63±0.24 | 0.43±0.22 |
|  | CH121 | 0.73±0.26 | 0.40±0.22 |

Note: The data presented are the mean ± standard deviation, n = 5.

**Table S11 Herbicide resistance of Bt-transgenic rice lines (BC_4_F_8_ and BC_4_F_9_) in the field**

| Year | Growth period | Lines | SPAD (Soil and Plant Analyzer Development) at the tillering stage | | | | | | | | | |
| --- | --- | --- | --- | --- | --- | --- | --- | --- | --- | --- | --- | --- |
|  |  |  | -3 | -2 | -1 | 1 | 2 | 3 | 4 | 5 | 6 | 7 |
| 2020  BC_4_F_8_ | Seedling stage | CHT025(1C) | 32.63±2.38 | 32.72±4.14 | 33.99±2.77 | 28.42±4.89 | 25.52±5.64 | 26.17±6.86 | 31.33±4.19 | 32.80±3.77 | 33.92±2.47 | 33.75±2.56 |
|  |  | CHT025(2A) | 32.49±2.78 | 32.95±2.59 | 32.65±2.74 | 27.29±6.16 | 28.19±7.89 | 32.62±4.77 | 26.97±6.64 | 33.55±2.11 | 30.76±7.89 | 30.76±7.89 |
|  |  | CH891(1C) | 31.81±2.54 | 32.80±3.37 | 34.56±2.16 | 28.66±4.36 | 29.55±5.37 | 30.63±4.37 | 32.70±4.96 | 31.26±7.31 | 34.87±3.26 | 33.47±1.67 |
|  |  | CH891(2A) | 32.59±2.74 | 32.88±3.19 | 32.27±2.97 | 28.99±6.24 | 28.50±6.24 | 25.83±5.41 | 25.83±7.19 | 33.47±1.67 | 32.31±6.86 | 32.31±6.86 |
|  |  | CH871(1C) | 32.79±2.20 | 32.16±4.31 | 34.80±2.78 | 27.70±5.50 | 27.70±5.50 | 32.64±2.72 | 32.64±2.72 | 33.35±3.95 | 33.36±1.98 | 33.52±2.09 |
|  |  | CH121(1C) | 31.95±2.28 | 32.93±3.35 | 34.48±1.99 | 28.49±4.73 | 27.66±5.69 | 29.61±7.17 | 29.59±8.20 | 30.02±8.91 | 34.67±2.75 | 33.55±2.11 |
|  |  | CHT025 | 32.02±1.80 | 32.60±2.92 | 32.87±2.87 | 26.77±3.05 | 24.73±3.13 | 20.89±5.42 | 17.32±4.58 | 13.78±3.76 | 12.79±2.70 | 11.43±2.60 |
|  |  | CH891 | 32.92±2.08 | 28.64±2.24 | 32.36±3.38 | 26.28±4.73 | 23.76±6.44 | 21.96±3.86 | 18.36±5.53 | 12.58±4.56 | 12.80±2.76 | 10.78±2.20 |
|  |  | CH871 | 31.34±1.92 | 27.76±3.90 | 32.18±2.91 | 27.90±1.67 | 26.44±3.48 | 16.86±2.55 | 15.54±2.49 | 13.38±3.67 | 11.94±3.61 | 9.88±2.48 |
|  |  | CH121 | 31.80±1.30 | 27.78±3.01 | 34.06±2.49 | 26.12±3.83 | 23.98±2.87 | 23.86±6.97 | 18.06±5.54 | 15.38±3.15 | 13.64±1.74 | 13.64±1.74 |
| 2021  BC_4_F_9_ | Seedling stage | CHT025(1C) | 32.31±2.54 | 32.48±4.50 | 34.53±2.83 | 27.52±4.62 | 26.28±5.72 | 27.11±3.14 | 31.23±4.69 | 32.11±2.97 | 32.64±2.84 | 32.31±2.59 |
|  |  | CHT025(2A) | 32.58±2.96 | 32.31±2.21 | 33.28±2.69 | 26.88±4.99 | 27.97±3.29 | 32.09±4.82 | 27.78±3.07 | 33.39±2.31 | 31.78±3.14 | 31.78±3.14 |
|  |  | CH891(1C) | 31.34±2.12 | 33.60±3.75 | 34.69±2.24 | 28.50±4.76 | 28.40±6.38 | 29.72±6.03 | 31.89±5.28 | 30.53±3.33 | 34.62±3.32 | 33.67±1.65 |
|  |  | CH891(2A) | 32.51±2.75 | 32.90±3.05 | 33.40±2.96 | 26.38±4.12 | 26.64±6.68 | 33.32±3.57 | 27.57±6.06 | 33.67±1.65 | 30.32±4.29 | 30.32±4.29 |
|  |  | CH871(1C) | 33.07±2.14 | 32.69±2.64 | 33.53±3.28 | 28.66±5.63 | 24.83±4.87 | 24.60±4.57 | 32.40±2.79 | 32.61±4.13 | 34.59±2.84 | 34.97±2.64 |
|  |  | CH121(1C) | 32.44±2.03 | 32.51±2.91 | 34.48±1.94 | 28.33±4.21 | 26.55±5.28 | 28.72±3.72 | 29.23±4.91 | 29.23±5.63 | 32.63±2.31 | 33.39±2.31 |
|  |  | CHT025 | 31.99±2.21 | 27.76±2.85 | 32.19±2.90 | 27.33±3.05 | 24.35±2.40 | 19.86±6.53 | 17.79±4.46 | 12.50±4.30 | 13.13±2.89 | 10.58±2.40 |
|  |  | CH891 | 32.48±1.65 | 28.01±3.23 | 33.55±2.78 | 26.52±3.29 | 24.78±3.50 | 22.28±5.17 | 21.07±4.94 | 20.23±4.14 | 18.98±2.83 | 18.01±2.83 |
|  |  | CH871 | 32.06±2.21 | 28.70±3.00 | 33.64±2.84 | 26.13±3.15 | 25.16±3.96 | 22.07±6.75 | 16.79±5.02 | 15.24±2.59 | 12.41±2.63 | 12.41±2.63 |
|  |  | CH121 | 31.52±1.49 | 27.21±3.04 | 33.40±2.96 | 26.48±3.27 | 25.87±2.63 | 18.88±5.28 | 16.83±5.12 | 13.73±4.15 | 12.09±2.64 | 11.22±2.43 |
| 2020  BC_4_F_8_ | Tillering stage | CHT025(1C) | 45.66±1.88 | 46.13±3.11 | 45.40±1.67 | 41.38±0.86 | 43.47±2.20 | 44.33±4.70 | 37.77±10.82 | 42.71±3.51 | 44.76±3.42 | 43.51±4.97 |
|  |  | CHT025(2A) | 48.48±4.77 | 49.31±4.92 | 48.89±2.45 | 44.87±2.59 | 36.78±5.99 | 32.36±9.35 | 41.78±4.86 | 40.06±1.98 | 42.74±3.74 | 42.81±7.27 |
|  |  | CH891(1C) | 46.24±1.40 | 46.50±3.63 | 44.59±2.58 | 43.43±5.47 | 36.66±4.13 | 44.10±3.10 | 45.79±2.12 | 44.47±3.85 | 44.08±3.58 | 44.98±3.48 |
|  |  | CH891(2A) | 51.00±3.27 | 48.04±2.95 | 49.31±3.01 | 44.68±3.52 | 36.94±5.03 | 38.28±6.50 | 40.28±5.81 | 42.92±5.09 | 44.71±5.42 | 43.02±6.88 |
|  |  | CH871(1C) | 48.16±3.63 | 48.67±3.35 | 47.37±3.87 | 40.06±5.00 | 39.23±9.82 | 25.21±10.88 | 35.79±10.11 | 39.98±5.52 | 42.53±6.14 | 40.78±10.31 |
|  |  | CH121(1C) | 52.22±5.84 | 49.83±5.08 | 49.06±4.41 | 41.38±6.36 | 35.12±5.41 | 41.21±4.19 | 47.37±3.87 | 49.31±3.01 | 45.66±4.13 | 45.68±6.89 |
|  |  | CHT025 | 46.34±2.12 | 50.29±3.97 | 48.99±3.76 | 41.41±6.88 | 26.46±7.61 | 30.66±3.20 | 29.87±4.24 | 12.34±6.52 | 9.92±5.91 | 12.92±1.25 |
|  |  | CH891 | 47.34±2.09 | 50.18±4.39 | 48.40±4.42 | 40.90±8.96 | 25.72±7.97 | 30.56±8.66 | 26.36±7.42 | 11.92±7.60 | 7.64±4.28 | 7.48±9.45 |
|  |  | CH871 | 48.90±5.59 | 47.62±3.24 | 47.67±3.31 | 37.53±6.10 | 23.98±5.69 | 18.72±5.77 | 20.61±8.59 | 12.36±6.60 | 10.23±5.29 | 9.74±3.94 |
|  |  | CH121 | 46.78±5.47 | 46.28±1.51 | 46.54±2.97 | 36.30±7.80 | 25.40±6.88 | 21.26±3.04 | 19.70±4.37 | 14.12±6.86 | 9.24±5.91 | 11.46±6.64 |
| 2021  BC_4_F_9_ | Tillering stage | CHT025(1C) | 42.58±5.58 | 50.88±2.90 | 41.96±1.97 | 42.88±2.74 | 30.68±6.90 | 27.62±4.46 | 40.54±3.08 | 42.06±3.47 | 46.64±4.28 | 46.18±4.42 |
|  |  | CHT025(2A) | 42.06±3.47 | 48.36±4.37 | 44.54±2.91 | 37.36±3.87 | 27.80±9.95 | 32.56±8.25 | 35.20±6.91 | 39.42±4.46 | 45.34±1.73 | 45.10±1.88 |
|  |  | CH891(1C) | 42.26±4.42 | 50.62±3.07 | 42.10±1.70 | 43.57±3.20 | 28.54±5.04 | 29.42±3.32 | 40.34±2.56 | 42.12±3.53 | 47.00±4.65 | 48.66±5.80 |
|  |  | CH891(2A) | 42.12±3.53 | 47.89±4.26 | 42.34±3.51 | 39.53±4.08 | 27.57±3.86 | 30.42±8.42 | 38.44±6.59 | 38.52±4.06 | 45.97±3.21 | 49.20±3.59 |
|  |  | CH871(1C) | 46.64±4.28 | 44.10±4.50 | 40.54±2.87 | 40.42±1.83 | 27.62±4.46 | 35.44±4.77 | 38.34±3.01 | 45.34±1.73 | 42.06±3.47 | 40.88±2.98 |
|  |  | CH121(1C) | 47.00±4.65 | 45.81±4.88 | 40.42±2.41 | 39.53±3.43 | 29.42±3.32 | 36.57±4.62 | 39.77±3.83 | 45.97±3.21 | 42.12±3.53 | 45.73±5.23 |
|  |  | CHT025 | 45.22±2.74 | 43.49±3.72 | 35.76±3.53 | 29.30±4.79 | 26.43±5.77 | 24.24±7.10 | 13.64±4.78 | 12.48±2.78 | 12.30±5.48 | 9.99±2.89 |
|  |  | CH891 | 45.50±2.59 | 44.37±5.92 | 39.97±2.37 | 29.40±2.81 | 30.27±3.85 | 18.63±4.45 | 12.83±6.59 | 11.43±4.05 | 8.63±3.00 | 8.03±2.74 |
|  |  | CH871 | 44.80±1.92 | 43.43±3.31 | 34.07±1.75 | 34.03±2.23 | 24.03±3.65 | 21.70±2.82 | 13.77±2.80 | 12.07±2.61 | 13.60±1.95 | 14.03±1.54 |
|  |  | CH121 | 45.37±4.38 | 42.67±2.68 | 33.23±0.90 | 24.47±3.19 | 25.00±3.10 | 32.40±4.16 | 15.43±2.56 | 13.93±1.62 | 14.67±1.09 | 16.50±0.96 |
| 2020  BC_4_F_8_ | Booting stage | CHT025(1C) | 44.36±4.06 | 42.44±4.16 | 44.36±4.06 | 41.36±4.37 | 35.89±3.70 | 26.79±9.19 | 34.25±9.36 | 37.13±2.21 | 39.14±5.37 | 39.99±4.38 |
|  |  | CHT025(2A) | 41.96±4.21 | 39.15±3.68 | 41.23±3.26 | 40.21±4.37 | 31.84±6.86 | 22.69±8.61 | 36.60±3.47 | 39.59±4.68 | 40.25±3.19 | 44.27±3.37 |
|  |  | CH891(1C) | 41.40±2.21 | 39.15±2.49 | 41.34±2.13 | 40.85±2.64 | 32.39±4.93 | 22.89±7.48 | 31.47±3.36 | 39.00±3.51 | 40.05±2.98 | 41.69±3.72 |
|  |  | CH891(2A) | 41.25±2.86 | 39.09±3.01 | 41.23±2.85 | 38.49±4.31 | 33.53±4.91 | 21.18±7.58 | 29.41±9.60 | 35.86±2.39 | 37.58±5.02 | 40.30±3.68 |
|  |  | CH871(1C) | 46.83±4.75 | 44.14±4.79 | 45.81±4.75 | 43.01±4.87 | 35.67±7.32 | 32.20±9.15 | 35.52±4.33 | 40.23±4.17 | 41.46±4.37 | 44.40±5.08 |
|  |  | CH121(1C) | 43.16±4.25 | 40.79±4.20 | 42.79±3.96 | 40.78±4.33 | 33.86±5.79 | 25.42±9.22 | 35.47±11.00 | 38.36±3.81 | 39.70±4.38 | 42.13±4.42 |
|  |  | CHT025 | 50.43±3.24 | 50.00±3.46 | 50.32±3.76 | 44.93±4.65 | 43.17±7.17 | 33.27±5.35 | 22.35±9.26 | 21.56±5.87 | 14.79±2.66 | 8.17±3.93 |
|  |  | CH891 | 46.31±3.00 | 45.53±2.44 | 47.93±3.79 | 43.21±5.53 | 39.67±7.81 | 26.41±6.80 | 23.55±9.66 | 21.11±6.59 | 13.57±4.67 | 8.87±3.49 |
|  |  | CH871 | 42.40±2.06 | 42.04±1.85 | 43.93±4.58 | 39.91±9.73 | 39.43±3.08 | 25.34±4.97 | 16.46±7.26 | 21.91±2.01 | 11.43±3.98 | 13.56±6.14 |
|  |  | CH121 | 46.38±4.30 | 45.86±4.19 | 47.39±4.75 | 42.68±7.07 | 40.76±9.54 | 28.34±2.77 | 20.79±3.08 | 21.53±2.14 | 13.26±3.98 | 10.20±5.12 |
| 2021  BC_4_F_9_ | Booting stage | CHT025(1C) | 49.15±3.67 | 44.63±4.55 | 50.23±3.40 | 42.23±1.64 | 35.89±3.70 | 36.36±4.37 | 40.15±6.43 | 39.14±5.37 | 39.99±4.38 | 46.66±4.38 |
|  |  | CHT025(2A) | 49.23±4.16 | 42.05±4.78 | 44.89±4.00 | 40.08±3.60 | 31.84±6.86 | 35.25±7.01 | 39.84±5.81 | 41.22±3.60 | 43.71±4.18 | 43.31±3.26 |
|  |  | CH891(1C) | 48.90±3.03 | 41.39±4.05 | 47.42±4.53 | 40.86±2.08 | 32.39±4.93 | 35.07±2.70 | 38.99±8.83 | 40.73±3.48 | 41.54±3.79 | 44.03±3.18 |
|  |  | CH891(2A) | 47.42±4.07 | 46.27±4.95 | 45.27±3.01 | 40.59±3.09 | 33.53±4.91 | 35.69±4.52 | 36.01±8.40 | 37.85±5.04 | 39.63±3.94 | 42.22±2.57 |
|  |  | CH871(1C) | 51.24±3.72 | 42.35±4.18 | 50.00±4.96 | 42.01±3.62 | 35.67±7.32 | 37.88±3.63 | 39.05±4.66 | 41.96±4.62 | 44.37±5.10 | 48.71±3.75 |
|  |  | CH121(1C) | 49.19±3.85 | 43.34±4.77 | 47.56±4.55 | 41.15±2.96 | 32.35±5.79 | 33.86±3.95 | 40.88±8.73 | 40.18±4.62 | 41.85±4.61 | 44.99±4.16 |
|  |  | CHT025 | 49.30±2.96 | 49.76±3.56 | 49.97±3.66 | 42.93±6.54 | 36.17±7.17 | 33.31±3.85 | 22.21±7.62 | 18.82±4.27 | 14.81±1.12 | 7.93±4.01 |
|  |  | CH891 | 49.16±5.13 | 45.95±2.39 | 46.87±4.31 | 41.75±6.34 | 35.67±7.81 | 27.93±7.87 | 21.29±7.43 | 15.61±7.64 | 13.86±4.15 | 9.44±2.83 |
|  |  | CH871 | 47.69±4.50 | 42.09±1.89 | 43.48±4.36 | 39.67±9.13 | 34.43±13.08 | 27.90±11.15 | 18.28±8.81 | 17.10±8.84 | 10.47±3.19 | 13.61±6.12 |
|  |  | CH121 | 48.72±4.21 | 45.93±4.12 | 46.77±4.80 | 41.45±7.31 | 35.76±9.54 | 29.71±8.28 | 20.59±7.91 | 17.18±7.06 | 13.05±3.53 | 10.33±5.01 |
| 2020  BC_4_F_8_ | Filling stage | CHT025(1C) | 44.47±3.96 | 41.63±1.74 | 39.21±2.23 | 38.01±3.27 | 30.47±6.60 | 27.49±3.51 | 33.63±3.24 | 37.74±3.77 | 41.25±2.38 | 44.47±3.96 |
|  |  | CHT025(2A) | 43.05±2.05 | 40.18±2.59 | 44.96±2.03 | 36.93±1.98 | 33.63±3.24 | 25.11±4.42 | 35.89±3.82 | 41.69±3.72 | 41.12±3.36 | 43.05±2.05 |
|  |  | CH891(1C) | 44.71±3.56 | 42.07±2.03 | 42.76±3.21 | 38.57±2.66 | 33.15±5.59 | 28.89±3.68 | 35.37±4.11 | 41.22±3.57 | 42.03±2.21 | 44.71±3.56 |
|  |  | CH891(2A) | 42.96±3.46 | 41.08±2.02 | 42.93±3.59 | 37.62±2.83 | 32.77±4.03 | 25.61±3.18 | 33.60±5.76 | 37.35±2.86 | 39.53±2.92 | 42.96±3.46 |
|  |  | CH871(1C) | 43.61±2.51 | 39.57±2.28 | 40.56±3.83 | 36.21±2.34 | 30.24±6.27 | 25.34±4.35 | 32.89±3.57 | 40.57±5.13 | 41.99±2.90 | 43.61±2.51 |
|  |  | CH121(1C) | 43.76±3.18 | 40.91±2.29 | 42.08±3.60 | 37.47±2.71 | 32.05±5.36 | 26.61±3.97 | 33.96±4.56 | 39.71±4.20 | 41.18±2.86 | 43.76±3.18 |
|  |  | CHT025 | 41.99±2.90 | 40.15±2.94 | 40.57±5.13 | 37.17±1.47 | 30.24±6.27 | 20.88±3.98 | 18.87±5.85 | 10.93±2.74 | 11.35±4.88 | 9.56±2.39 |
|  |  | CH891 | 42.03±2.21 | 40.29±1.94 | 41.22±3.57 | 36.78±3.02 | 33.15±5.59 | 21.02±4.28 | 17.99±4.04 | 9.86±2.76 | 11.73±3.42 | 8.39±2.67 |
|  |  | CH871 | 39.53±2.92 | 38.16±3.31 | 37.35±2.86 | 36.3±2.50 | 32.77±4.03 | 19.26±4.73 | 15.60±2.16 | 9.41±2.24 | 10.26±3.35 | 8.27±2.27 |
|  |  | CH121 | 41.18±2.86 | 39.53±2.87 | 39.71±4.20 | 36.75±2.36 | 32.05±5.36 | 20.39±4.25 | 17.49±4.39 | 10.07±2.77 | 11.11±3.14 | 8.74±2.43 |
| 2021  BC_4_F_9_ | Filling stage | CHT025(1C) | 41.27±2.38 | 41.79±2.24 | 40.51±2.33 | 39.03±2.62 | 33.15±5.36 | 27.95±3.86 | 34.53±2.75 | 38.36±3.41 | 40.65±2.34 | 44.95±3.74 |
|  |  | CHT025(2A) | 41.12±3.36 | 41.1±2.36 | 45.33±2.76 | 38.05±2.52 | 33.97±2.90 | 26.83±4.16 | 36.14±3.46 | 40.03±3.51 | 39.91±2.98 | 42.73±1.76 |
|  |  | CH891(1C) | 42.03±2.21 | 41.08±2.02 | 42.93±3.59 | 37.62±2.83 | 32.77±4.03 | 25.61±3.18 | 33.60±5.76 | 37.35±2.86 | 39.53±2.92 | 42.96±3.46 |
|  |  | CH891(2A) | 39.53±2.92 | 41.19±2.83 | 43.07±4.05 | 39.44±2.11 | 34.75±2.88 | 28.51±3.82 | 33.88±4.41 | 39.01±3.20 | 39.28±2.06 | 43.84±2.09 |
|  |  | CH871(1C) | 41.99±2.90 | 42.07±2.03 | 42.76±3.21 | 38.57±2.66 | 33.15±5.59 | 28.90±3.68 | 35.37±4.11 | 41.22±3.57 | 42.03±2.21 | 44.71±3.56 |
|  |  | CH121(1C) | 41.18±2.86 | 41.45±2.29 | 42.92±3.51 | 38.54±2.57 | 33.56±4.25 | 27.67±3.74 | 34.29±4.73 | 39.19±3.50 | 40.28±2.66 | 43.84±3.09 |
|  |  | CHT025 | 41.35±4.88 | 40.29±1.94 | 41.22±3.57 | 36.78±3.02 | 33.15±5.59 | 21.02±4.28 | 17.99±4.04 | 9.86±6.76 | 11.73±6.42 | 8.39±5.67 |
|  |  | CH891 | 41.73±6.42 | 38.16±3.31 | 37.35±2.86 | 36.3±2.50 | 32.77±4.03 | 19.26±4.73 | 15.6±2.16 | 9.41±5.24 | 10.26±4.35 | 8.27±3.27 |
|  |  | CH871 | 40.26±4.35 | 38.07±3.85 | 39.01±3.20 | 35.15±2.79 | 34.75±2.88 | 18.11±3.72 | 17.13±2.32 | 7.55±2.00 | 7.97±2.06 | 8.77±3.68 |
|  |  | CH121 | 41.11±5.14 | 38.84±3.20 | 39.19±3.50 | 36.08±2.77 | 33.56±4.25 | 19.46±4.27 | 16.91±3.04 | 8.94±5.00 | 9.99±4.74 | 8.48±4.19 |

Note: The data presented are the mean ± standard deviation, n = 5.

**Table S12 Yields and relative traits of Bt-transgenic rice lines (BC_4_F_8_ and BC_4_F_9_) and their respective non-transgenic counterparts under pesticide-free environment**

| Year | Lines | Panicles per plant | Grains per panicle | Weight per 1000-grain | Yield per plant |
| --- | --- | --- | --- | --- | --- |
| 2020  BC_4_F_8_ | CHT025(1C) | 6.33±1.02 a | 262.28±15.69 c | 20.72±0.00 b | 34.41±0.58 b |
|  | CHT025(2A) | 11.00±0.29 c | 154.58±1.87 c | 22.96±0.01 c | 38.96±2.00 c |
|  | CH891(1C) | 7.67±0.56 b | 133.81±7.34 a | 26.39±0.05 b | 26.87±2.89 b |
|  | CH891(2A) | 6.67±0.18 b | 168.46±5.47 c | 22.88±0.07 c | 25.70±2.08 b |
|  | CH871(1C) | 9.50±0.01 c | 178.63±9.31 b | 21.23±0.02 a | 35.26±3.54 b |
|  | CH121(1C) | 8.67±0.15 b | 124.12±3.81 a | 29.43±0.04 b | 31.66±1.53 b |
|  | Changhui T025 | 6.50±0.71 a | 206.12±4.39 a | 18.55±0.03 a | 24.85±0.71 a |
|  | Changhui 891 | 5.33±1.37 a | 124.66±3.06 a | 28.02±0.05 a | 18.63±0.58 a |
|  | Changhui 871 | 5.33±0.46 a | 203.17±14.91 a | 22.05±0.06 a | 23.89±0.58 a |
|  | Changhui 121 | 6.67±0.32 a | 137.67±6.30 a | 25.21±0.03 c | 23.02±0.58 a |
| 2021  BC_4_F_9_ | CHT025(1C) | 8.67±0.36 b | 211.06±4.69 c | 18.78±0.03 c | 34.08±1.15 b |
|  | CHT025(2A) | 7.33±0.15 b | 211.06±6.68 c | 19.96±0.00 c | 39.18±1.53 c |
|  | CH891(1C) | 7.00±0.12 a | 169.61±5.82 c | 23.02±0.02 b | 27.50±1.00 b |
|  | CH891(2A) | 8.67±0.18 b | 136.74±5.47 b | 21.44±0.07 c | 25.76±2.08 b |
|  | CH871(1C) | 9.00±0.22 b | 188.52±9.10 c | 22.88±0.03 c | 39.07±1.73 c |
|  | CH121(1C) | 8.33±0.60 b | 146.84±4.59 a | 26.65±0.03 b | 32.61±0.58 b |
|  | Changhui T025 | 6.33±0.48 a | 145.58±5.73 a | 23.23±0.01 a | 21.42±0.58 a |
|  | Changhui 891 | 7.33±0.28 a | 117.25±3.11 a | 25.19±0.05 a | 22.04±0.58 a |
|  | Changhui 871 | 6.50±1.23 a | 128.60±15.52 a | 26.03±0.02 a | 21.75±0.71 a |
|  | Changhui 121 | 5.00±0.58 a | 141.35±2.49 a | 28.92±0.09 a | 20.44±2.65 a |
| ANOVA | Y | ns | * | ns | ns |
|  | G | * | * | * | * |
|  | L*G | * | * | * | * |

Note: The comparison of different Year and different Genetype were carried out yields relative traits of Bt-transgenic rice lines (BC_4_F_8_ and BC_4_F_9_) and their respective non-transgenic counterparts under pesticide-free environment. Data followed by different lower-case letters denote significant differences between genotypes at the 5% level according to LSD test. * represents the significant difference at the 5% level according to LSD test, ns represents no significant difference.


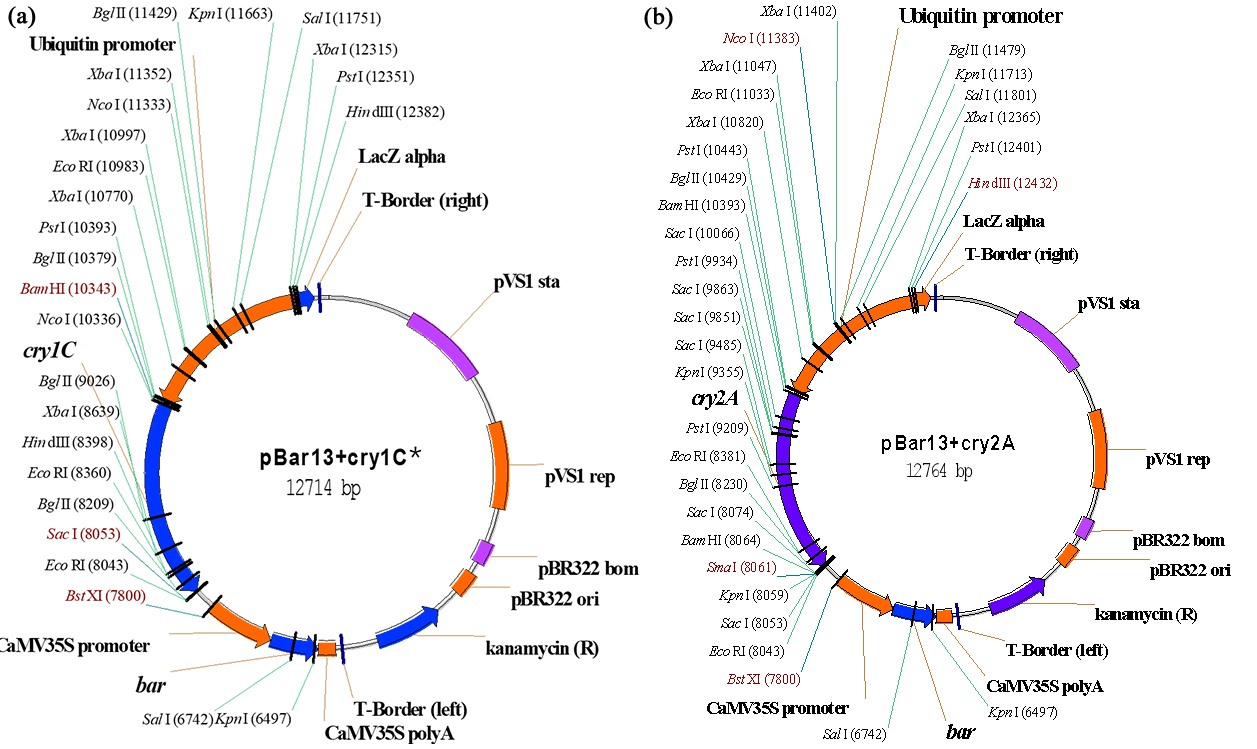


**Figure S1. Construction of *CRY1C*, *CRY2A* and *BAR* vectors.** The *CRY1C* and *CRY2A* gene was driven by a Ubiquitin promoter and terminated by the nopaline synthase (Nos) terminator. The *BAR* gene was also used as a selectable marker and was under the control of the CaMV35S promoter and tailed by the CaMV35S polyA. T-Border (left): left border of T-DNA region; T-Border (right): right border of T-DNA region.
